# The microbiomes of blowflies and houseflies as bacterial transmission reservoirs

**DOI:** 10.1038/s41598-017-16353-x

**Published:** 2017-11-24

**Authors:** Ana Carolina M. Junqueira, Aakrosh Ratan, Enzo Acerbi, Daniela I. Drautz-Moses, Balakrishnan N. V. Premkrishnan, Paul I. Costea, Bodo Linz, Rikky W. Purbojati, Daniel F. Paulo, Nicolas E. Gaultier, Poorani Subramanian, Nur A. Hasan, Rita R. Colwell, Peer Bork, Ana Maria L. Azeredo-Espin, Donald A. Bryant, Stephan C. Schuster

**Affiliations:** 1 Present Address: Singapore Centre for Environmental Life Sciences Engineering, Nanyang Technological University, Singapore, 637551 Singapore; 2 Department of Public Health Sciences and Center for Public Health Genomics, University of Virginia, Charlottesville, VA 22908 USA; 3 European Molecular Biology Laboratory, Structural and Computational Biology Unit, Heidelberg, 69117 Germany; 4 Center for Vaccines and Immunology, College of Veterinary Medicine, University of Georgia, Athens, 30602 GA USA; 5 Centro de Biologia Molecular e Engenharia Genética, Departamento de Genética, Evolução e Bioagentes, Instituto de Biologia, Universidade Estadual de Campinas, Campinas, SP 13083-875 Brazil; 6 CosmosID Inc, Rockville, MD 20850 USA; 7 Center for Bioinformatics and Computational Biology, University of Maryland. Institute for Computational Biology, University of Maryland College Park, College Park, MD 20742 USA; 8 Johns Hopkins Bloomberg School of Public Health, Baltimore, MD 21205 USA; 9 Department of Biochemistry and Molecular Biology, The Pennsylvania State University, University Park, PA 16802 USA; 10 Departamento de Genética, Instituto de Biologia, Universidade Federal do Rio de Janeiro, Rio de Janeiro, RJ 21941-902 Brazil

## Abstract

Blowflies and houseflies are mechanical vectors inhabiting synanthropic environments around the world. They feed and breed in fecal and decaying organic matter, but the microbiome they harbour and transport is largely uncharacterized. We sampled 116 individual houseflies and blowflies from varying habitats on three continents and subjected them to high-coverage, whole-genome shotgun sequencing. This allowed for genomic and metagenomic analyses of the host-associated microbiome at the species level. Both fly host species segregate based on principal coordinate analysis of their microbial communities, but they also show an overlapping core microbiome. Legs and wings displayed the largest microbial diversity and were shown to be an important route for microbial dispersion. The environmental sequencing approach presented here detected a stochastic distribution of human pathogens, such as *Helicobacter pylori*, thereby demonstrating the potential of flies as proxies for environmental and public health surveillance.

## Introduction

Interactions between hosts and microorganisms are increasingly recognized as a ubiquitous principle in nature^1^, capable of modulating animal physiology, fitness, and host social behaviour^2^. Most of the microbiome research has been performed in humans, but massively parallel sequencing and recent advances in computational analyses have made it possible to extend the same level of taxonomic resolution to hosts from other phyla. More recently, mammals^3^, plants^4^, and insects^5^ were also targeted by metagenomics. In insects, the microbiomes of termites, bees, ants, flies, mosquitoes, and the triatome bug^6^ have recently been analysed. However, many of these studies have been restricted to 16S sequencing^7^, which generally limits the identification of microbial taxa by the experimental shortcomings and biases of PCR-based and/or cultivation-based methods, yet providing a record of the phylum- and genus-level diversity.

Despite their medical, forensic, and sanitary importance, an assessment of the microbiome of mechanical vectors, such as blowflies (Diptera: Calliphoridae) and houseflies (Diptera: Muscidae), has not been undertaken by large-scale metagenomic approaches. Although transmission of microbiota between hosts has been reported between humans and pets in a household^8^, potential interchange of the microbiota of synanthropic insects and their surroundings is under-characterized.

Blowflies and houseflies are the first organisms to arrive on carcasses, decaying organic matter and faeces on which they feed, breed, and lay eggs^9^. It is likely that they acquire a significant part of their microbiome from such environments, which they transport and subsequently deposit onto other hosts, such as humans, animals, and plants. Because they are ubiquitous and synanthropic, these flies play an important role in the transport and dispersal of microbes in urban and natural environments. It has been suggested that flies serve as a vector for a number of pathogens^10,11^, but they have rarely been implicated in any specific medical condition. This absence of cause-effect linkage has prevented more targeted studies of mechanical vectors, compared to biological vectors, for which host-microbe associations have been intensively analysed^12^.

In this study, we have investigated 116 microbiomes by whole-genome shotgun (WGS) sequencing of 63 samples of blowflies of the species *Chrysomya megacephala* and 53 individual houseflies of the species *Musca domestica*, describing the complexity of the pan-microbiomes of both mechanical vectors at the species level. Flies were collected on three continents in urban, rural, and natural settings. Despite inhabiting similar ecological niches, they showed different compositions and abundances for the organisms in their associated microbial communities. Host-specific microbial taxa were observed, but blowflies and houseflies share more than 50% of their microbiome that is likely acquired from similar habitats where they feed and breed. To depict the microbiomes of both fly species, we analysed the entire dataset using three approaches with different degrees of stringency, which we combined for high confidence of taxa assignment. This allowed us to reach a species-level identification of the microbial communities in these mechanical vectors and thus provide a better understanding of the roles of these flies as potential agents of pathogen transmission.

## Results and Discussion

### Sampling and environmental sequencing approach

**Figure 1:**
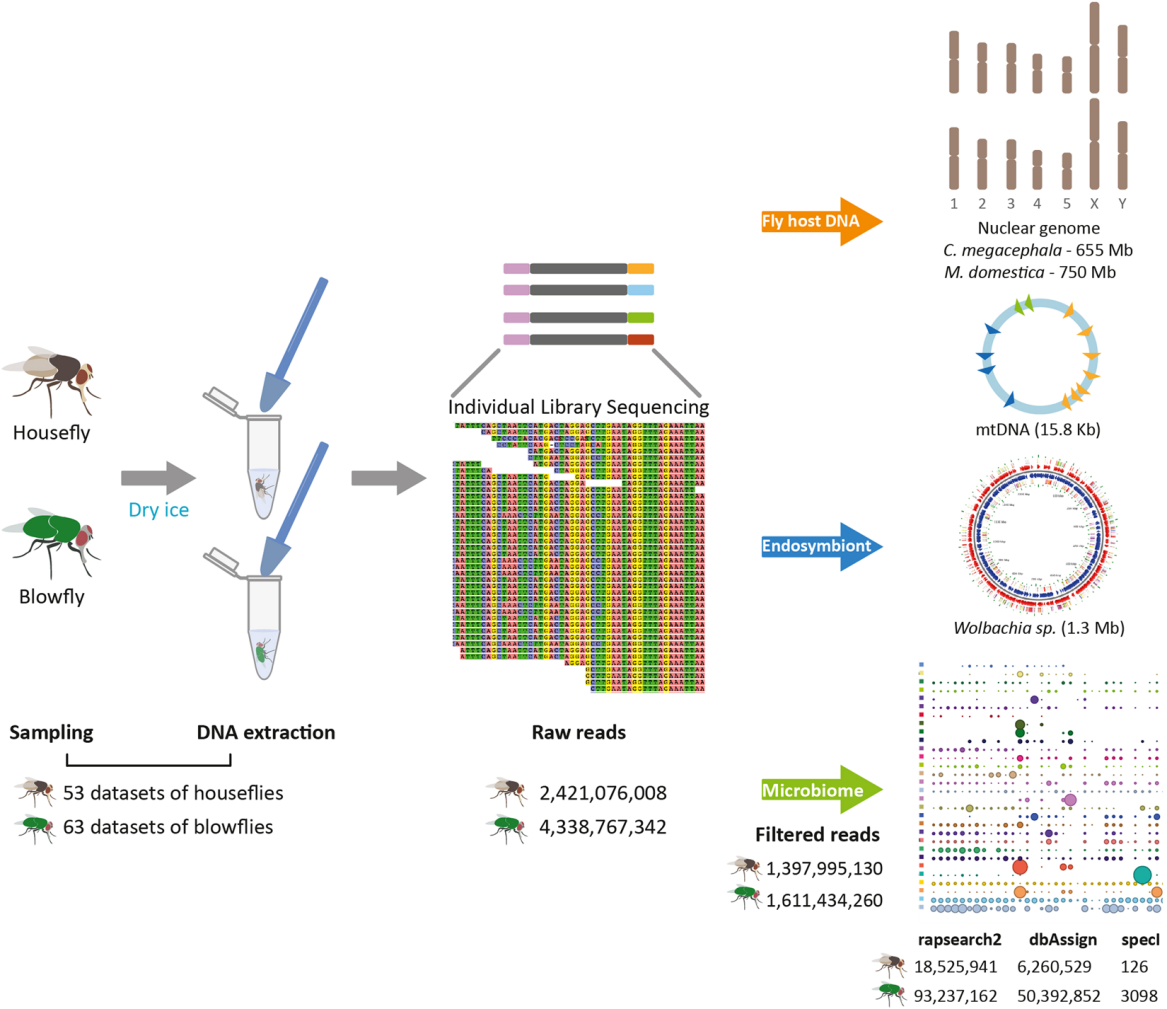
Summary of sampling datasets, data generation and analyses. Blowflies (n = 62; 1 control) and houseflies (n = 53) were collected in individual vials and immediately placed on dry ice until DNA extraction. Samples were individually sequenced in a multiplexed run, generating a total of 6,759,843,350 reads for both fly species. The blowfly draft genome generated in this study and the housefly reference genome (RefSeq number GCF_000371365.1) were used as filters to remove host-related reads. Final metagenomic dataset included a total of 3,009,429,390 reads for 116 flies. See also Tables S1 for a summary of reads generated and assigned to blowflies and houseflies, and Table S2 for the detailed information of each individual sample. Reads were processed with three different bioinformatics methods and assigned to bacterial taxa using the rapsearch2 algorithm against the NR database (April 2015 version), the dbAssign in-house script (https://github.com/aakrosh/dbAssign) against a database with 5,614 complete and chromosome-level assembled microbial genomes (April 2016 version) and a BWA approach against specI clusters (Tables S3, S4 and S5 for detailed information).

Blowflies and houseflies were caught in different environments on three continents using a protocol that prevented sample contamination while preserving nucleic acids found in or on the insect body. Cross-contamination was avoided by using dry-ice, which immediately suffocated the animals and ensured instant preservation of the entire specimen at low temperature at the sampling sites under sterile conditions (see Material and Methods). Using the entire body of a fly, a novel metagenomic approach was used to sequence DNA molecules of the insect and its microbiome together. Therefore, individual flies may serve as environmental proxies that provide information on the locations they have recently visited. We believe this versatile approach could be applicable to environmental and public health surveillance as well as forensics. In contrast to previous studies that focused only on cultivatable microorganisms, insect midguts or 16S rRNA^7,13,14^, our metagenomics strategy resulted in coverage of the host and microbiome genomes. Specifically, the genomes of *M*. *domestica* and *C*. *megacephala* were sequenced to a depth of 3.2-fold and 6.6-fold respectively, the host mitochondrial DNA (mtDNA) was sequenced to a depth of 7000-fold^15^, and the *Wolbachia* spp. endosymbiont genome was covered to a depth of >2000-fold. The remaining >93 million reads were effectively assigned to the microbiomes of the respective hosts (Fig. 1).

### Microbial assignment of the metagenomic datasets

**Figure 2:**
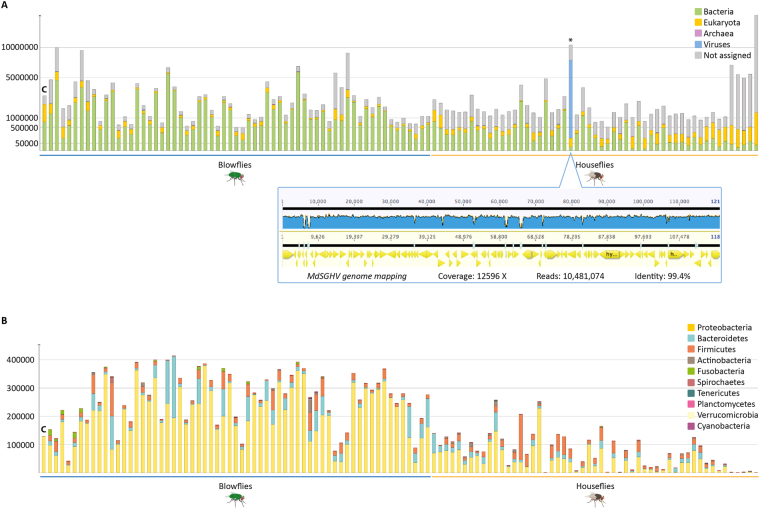
Higher rank taxonomy of the microbiome of blowflies and houseflies. (**A**) Super kingdom classification of the metagenomic reads, indicating bacteria are the main component of the microbiome of fly mechanical vectors. Reads assigned to Eukaryota are mostly assigned to insects (Diptera, in particular. See Supplementary Figure S1 for detailed analysis of the eukaryote reads). The sample marked with an * shows a high virus load that was identified as the MdSGHV DNA virus that infects houseflies. The genome mapping of viral reads against the MdSGHV reference genome showed that the metagenomic dataset was spread across the viral genome with >12,000-fold coverage on average. (**B**) Bacterial counterpart of the metagenomic reads at phylum-level taxonomic rank. *Proteobacteria* dominates the microbiome of blowflies and houseflies, followed by *Bacteroidetes* and *Firmicutes*. Most of the proteobacterial reads are assigned to the endosymbiont *Wolbachia sp*. in blowflies and to *Psychrobacter sp*. PRwf-1 in houseflies. Detailed results also show the presence of the *Wolbachia* endosymbiont in housefly samples collected in three different countries. Sample marked with “C” indicates the lab-reared pool sample serving as a control.

We generated a total of 116 separate metagenomic datasets (blowflies = 62; houseflies = 53; lab-reared pooled control = 1) from 3 different continents. The blowfly datasets contained approximately 70 million reads per sample (control excluded) and the housefly datasets had approximately 45 million reads per sample (Table S1 for an average of reads per sample). A total of 6,759,843,350 reads were generated. After the *in silico* removal of the fly genomic sequences using Bowtie2^16^, the remaining 3,009,429,390 reads (44%; Fig. 1 and Table S1) were used for downstream metagenomics analyses with three different bioinformatics methods: (1) rapsearch2, (2) dbAssign and (3) specI (Table S1 for summary, Table S2 for extended information). When collapsed into super kingdom taxonomy (Fig. 2A), these large-scale datasets showed minimal traces of Archaea. Most of the reads assigned to Eukaryotes belong to the order Diptera, indicative of the incompleteness of the reference genome for these species (Figure S1). Sequences assigned to the domain Bacteria are the most prevalent in all datasets, except in the housefly sample AJ155 (identified with an asterisk on Fig. 2A), in which viral DNA was highly abundant. An in-depth analysis of this sample revealed the presence of the *M*. *domestica* Salivary Gland Hypertrophy Virus (MdSGHV). The alignment of viral reads against the MdSGHV reference genome^17^ (NC_01067) gave a mean coverage of 12,596-fold (detailed in Fig. 2A). MdSGHV is a double-stranded DNA virus that is orally transmitted to houseflies and causes the inhibition of ovarian development, thus leading to a shutdown of egg production in infected females. Flies also show hypertrophy of the salivary gland as a symptom^18^. The other viruses observed in these datasets were mainly bacteriophages (Figure S2).

**Figure 3:**
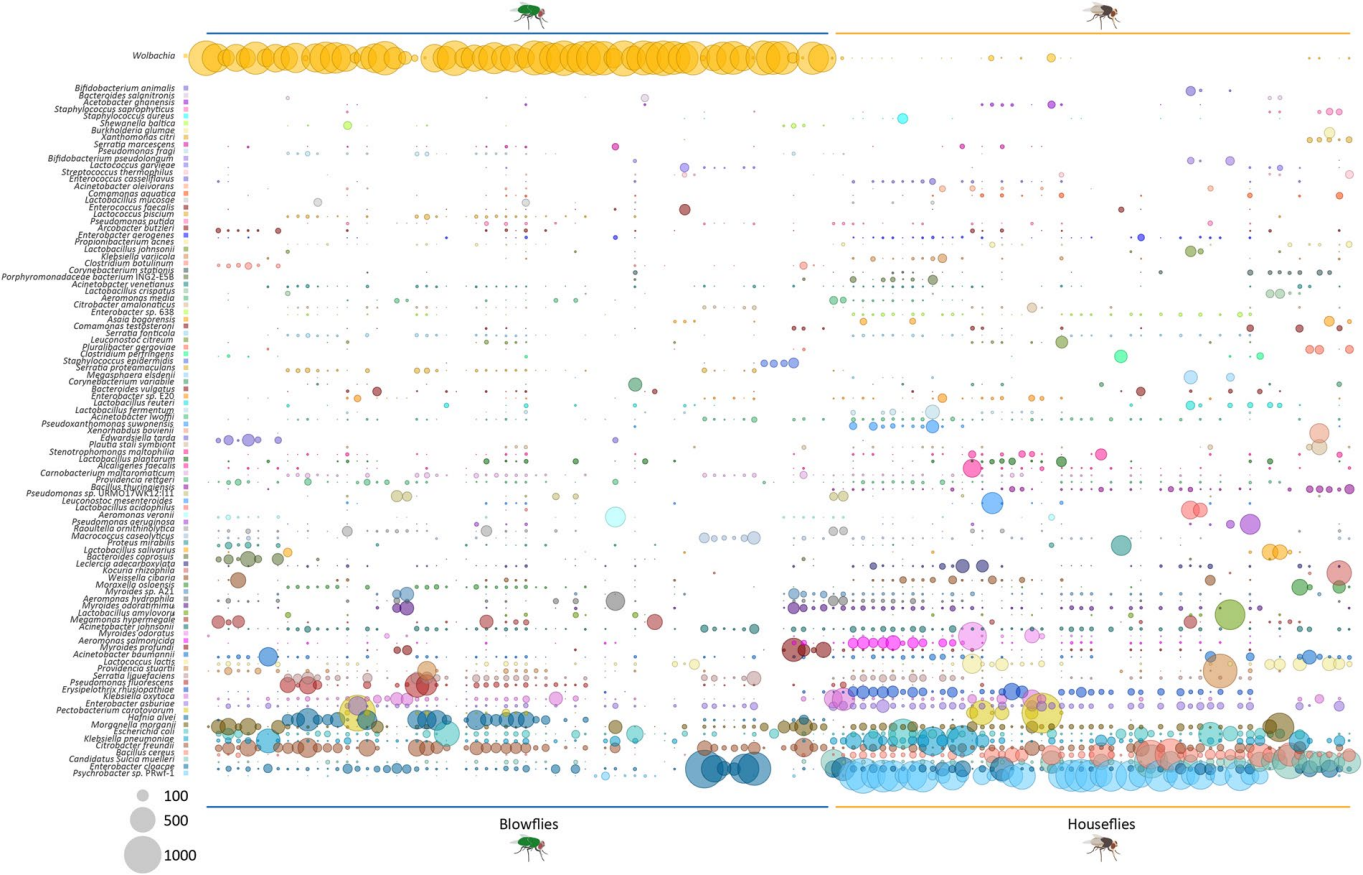
Microbiome of blowflies and houseflies. The bubblechart shows the top 100 bacterial species assigned to 116 fly sample analysed by the dbAssign tool kit. The size of the bubbles is square-root-scaled (scale in grey) and refers to the normalized number of reads assigned to each bacterial reference genome (listed in the Y axis) in each of the samples (listed in the X axis). See Figure S4 for alpha-diversity indices.

Taxa assignments were performed with normalized datasets (see Methods), which showed that members of the phyla *Proteobacteria*, *Bacteroidetes* and *Firmicutes* are the most abundant organisms in the microbiomes of both blowflies and houseflies (Fig. 2B and Figure S3). This result corroborates previous findings for the green bottle fly^7^, houseflies^19^, bees, cockroaches, fruit flies and mosquitoes^20^, except for the low representation of *Actinobacteria* in our datasets. This difference is likely due to that fact that insect studies use cultivation and amplification of the 16S rRNA, while this study was undertaken with amplification-free metagenomics based on WGS. The preponderance of *Proteobacteria* in blowflies is mainly associated with the presence of the endosymbiont *Wolbachia* (Alphaproteobacteria), while in houseflies it is related to the dominance of *Psychrobacter* (Gammaproteobacteria). However, other members of the *Proteobacteria* are also major components of the blowfly and housefly microbiomes, including the genera *Enterobacter*, *Escherichia*, *Klebsiella*, *Proteus*, *Morganella*, *Hafnia*, *Pseudomonas*, *Aeromonas*, *Acinetobacter*, *Providencia* and *Serratia* (Fig. 3). Some of these bacterial genera have previously been identified by cultivation or amplification assays in different species of flies^7,21^.

The DNA-protein (rapsearch2) and DNA-DNA (dbAssign and specI) alignment approaches allowed species-level identification of the bacteria in the microbiome (Fig. 3 and Tables S3, S4 and S5 for the normalized number of reads assigned by dbAssign, rapsearch2 and the specI, respectively). All three methods showed that the prevalent microorganisms associated with the blowfly *C*. *megacephala* are similar to the endosymbionts *Wolbachia pipientis* of *Culex quinquefasciatus* and *Wolbachia* sp. *wRI* (isolated from *Drosophila simulans*). Both *Wolbachia* strains were collapsed into *Wolbachia* spp. in Fig. 3 and represent 60.9%, 62.5% and 87.1% of the total bacterial assigned reads analysed with rapsearch2, dbAssign and the specI pipelines, respectively. The broad distribution of *Wolbachia* spp. in all *C*. *megacephala* samples analysed confers a higher read count of bacteria in blowflies than houseflies (Fig. 2A and B). The genus *Wolbachia* is one of the most pandemic bacterial genera, described in more than 60% of insect species worldwide^22^ and its presence in the blowfly *C*. *megacephala* has previously been reported^23^. However, unlike former studies, our analysis not only indicates the presence of the endosymbiont, but simultaneously allowed the quantitative assessment of the relative distribution of *Wolbachia* across the samples. A total of 31,883,141 reads were assigned to the *Wolbachia* endosymbiont in blowfly samples, which provided >2000-fold coverage of its genome. The pairwise identity of the *Wolbachia* surface protein gene (*wsp*), commonly used for typing *Wolbachia* strains^24^, shows that *C*. *megacephala* and the butterfly *Eurema hecabe* (AB094396.1) have strains with 100% identity. However, the genome-wide mapping of assigned reads indicates similarity to the *Wolbachia* sp. from the mosquito *Culex quinquefasciatus* (NC_010981.1). Based on these lines of evidence, we hypothesize that *C*. *megacephala* hosts a specific strain of *Wolbachia* classified in the supergroup B branch. This intracellular symbiont plays important roles in manipulating invertebrate reproductive biology by killing males, feminization, parthenogenesis or cytoplasmic incompatibility, and it has been extensively studied as a biological control agent for arthropods^22^. Evidence of horizontal transmission of *Wolbachia* within and between species has been reported^22^ and, more recently, it was also shown that some *Wolbachia* spp. strains can influence the resistance of different hosts to viruses^25^.

In houseflies, there are sparse reports of *Wolbachia* spp. infection^23,26^ and this is the first unbiased report of the bacteria in houseflies, that is, without specific cultivation or PCR amplification (Fig. 3). A total of 4,016 reads were assigned to the *Wolbachia* endosymbiont in 29 housefly samples. The reads covered 23.8% (353,027 bp) of the *Wolbachia* sp. reference genome (NC_010981) with a pairwise identity of 99.2%. However, the strain could not be identified due to low coverage of both the genome and the *wsp* gene.

After subtracting reads that map to fly or mitochondrial DNA, we analysed the remaining microbial read abundances. The most prevalent bacterium detected in the microbiome of houseflies is the *Psychrobacter* sp. PRwf-1, present in 73.6% of the individuals analysed (Fig. 3) and represents 9.5%, 2.4% and 1.5% of all reads assigned by dbAssign, rapsearch2 and specI, respectively. Members of the genus *Psychrobacter* are extremophilic bacteria associated with cold environments and permafrost soils with remarkable capabilities to adapt to subzero temperatures. However, the strain PRwf-1 that accounts for 25% of the housefly microbiome (Fig. 3), shows physiological adaptation to survival in warmer temperatures and has been previously associated with food spoilage^27^.

Figure 3 compiles the top 100 bacterial species (vertical axis) assigned to each individual fly (horizontal axis) using dbAssign, representing 97.3% of all reads assigned. The normalized datasets of 116 flies resulted in the identification of 431 bacterial taxa at the species-level (Table S3). The species distribution patterns seen in Fig. 3 suggest that blowflies and houseflies share parts of their microbiomes, but also have host-specific microbial occurrences. The alpha-diversity calculated for rapsearch2 and dbAssign (Figure S4) shows that these values depend on the reference database composition and the stringency used for and after the alignment of short-read sequences to these databases. Rapsearch2, based on the translated nucleotide sequence similarity search against the NR protein database, shows a slightly higher microbial diversity of blowflies when compared to houseflies (median of 5.18 and 5.04, respectively), while the dbAssign analysis, based on the assignment of reads to reference genomes in a database, showed the opposite (blowflies = 1.69; houseflies = 3.15). The OTU (operational taxonomic unit) richness analysis showed an average number of 551 ± 373 bacterial species for blowflies and 228 ± 157 bacterial species for houseflies using rapsearch2 (Figure S5), and 37 ± 22 OTUs for blowflies and 42 ± 20 OTUs for houseflies using dbAssign (Figure S5). Species richness and sequence depth can also be evaluated with rarefaction curves (Figure S5), which showed that sequence depth plateaus at 25,000 reads for rapsearch2 and around 500 reads for dbAssign. These findings underline the importance of database size and diversity for metagenomic analyses and affected both approaches used in this study (nr database = 131,807,364 records; full microbial genomes database = 5,614 records).

### Newly emerged lab-reared flies as an environment control

The control sample comprised of a pool of 98 newly emerged adults of blowflies (*C*. *megacephala*) reared in cages under controlled conditions. The pool of adults showed a low alpha-diversity (3.0 for rapsearch2 and 0.58 for dbAssign) and is mainly composed of *Wolbachia sp*., with 99.5% of the microbiome reads assigned to the endosymbiont. Only four additional taxa are present in the control: *Morganella morganii*, *Myroides odoratus*, *Providencia rettgeri*, and *Providencia stuartii*, all with very low read counts. The reduced microbiome is likely due to the fact that holometabolous insects (i.e. those undergoing complete metamorphosis) expel their degenerated larval and pupal guts soon after eclosion^28^. They also replace the cuticle that will develop into an exoskeleton^29^. These developmental changes may eliminate not only the gut bacteria, but also those attached to the outer body surface, thus substantially reducing the endogenous fly microbiome diversity and complexity. The gut microbiomes of newly emerged adults were also of similarly low complexity for different mosquito species and worker bees of *Apis mellifera* ^30,31^.

### Shared and unique microbiomes

Using dbAssign, 316 bacterial taxa were identified in blowflies, compared to 351 in houseflies (Table S6). A comparison of the summarized microbiomes of houseflies and blowflies showed that more than 55% (237 taxa) of the microbial species are shared between the two hosts (Figure S6 and Table S6). The large overlap between microbiomes is likely related to the similar environments that both species frequent, from which they acquire a major part of the microorganisms they transport. The analysis of the shared microbiome showed that seven taxa are present in at least 80% of all flies analysed (Figure S7), including *E*. *coli* and *Enterobacter cloacae* that could be detected at the species level. Both species are gram-negative, facultatively anaerobic bacteria that are found as part of the normal gut flora of humans and animals. However, strains of *E*. *cloacae* can play an important role in nosocomial infections of the urinary and respiratory tract^32^, while *E*. *coli* serotypes are typically responsible for food poisoning and gastrointestinal infections^33^. All other microbial taxa of the core microbiome could be assigned only to genus level or a higher taxonomic rank. This includes the genera *Klebsiella* and *Acinetobacter* and additional organisms classified at the class, order or family level (Figure S7).

**Figure 4:**
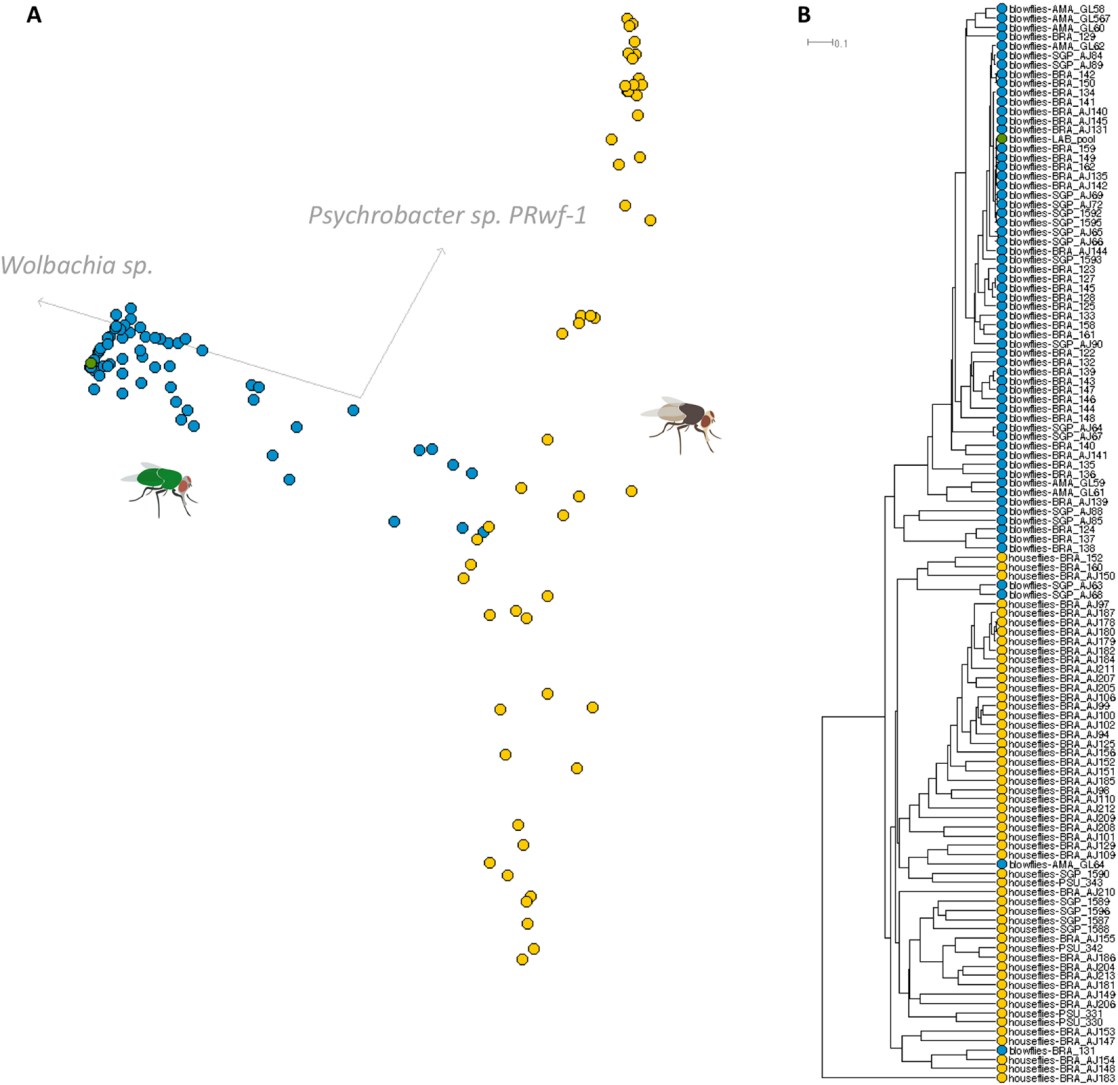
Segregation of fly host species based on their microbiome. (**A**) PCoA using Bray-Curtis index (stress = 0.12) showing that *C*. *megacephala* (blue dots) and *M*. *domestica* (yellow dots) individuals can be separated based on their full microbiome complexity, despite the fact that they share >50% of their microbiome (see also Figure S6, Figure S7, and Table S6). A few blowflies clustered with housefly individuals and deeper analyses showed an abnormal low amount of *Wolbachia* sp. endosymbiont in these blowflies. (**B**) UPGMA dendogram visualization of the clustering analysis, showing the samples of houseflies and blowflies that clustered together. The detailed microbiome profiles of samples GL64, BRA_131, AJ63 and AJ68 are shown in Figure S8.

Despite the fact that blowflies and houseflies share more than half of their microbiomes, host-species specific bacterial taxa were also detected with a cutoff of a minimum of 500 reads per species. A total of 114 microbial species were uniquely found in houseflies and 79 in blowflies. The host-specific microbiome is most likely driven by the fly species, but may also occur as a result of stochastic events. As shown in Fig. 3, random occurrence of microbial taxa can be observed in one or a few individuals of flies at significant numbers. In fact, principal coordinate analysis (PCoA) show segregation of the two fly species, with minimal overlap, based on their microbiomes (Fig. 4). The few blowfly individuals with microbiomes that overlap with those of houseflies display a remarkably low abundance of *Wolbachia* spp., as well as higher read counts that were assigned to *Enterobacter cloacae*, *Klebsiella pneumoniae* or *Pectobacterium carotovorum* (Figure S8).

To test the effect of geographic location on the observed variation of the microbiome, analysis of variance (ANOVA) and permutational multivariate analysis of variance (PERMANOVA) were performed. ANOVA indicates no significant discrimination of the microbiome of flies collected in different countries (p > 0.05). PERMANOVA suggests that the geographic origin has a minor effect on the beta-diversity of the microbiome (R^2^ = 0.057, p-value = 0.002 for rapseach2; R^2^ = 0.074, p-value = 0.001 for dbAssign), thus explaining a small proportion of the variation observed. The PCoA plots in Figure S9 show that samples from different continents do not segregate and that most of the microbiome variation is likely found between individuals and not between populations.

### Intersection of three metagenomic analyses

The complete assignment of reads to microbial taxa, using the three bioinformatic methods employed in this study, is shown in Tables S3, S4 and S5. The number of OTUs assigned to species level by each method is summarized in the Venn diagram in Figure S10 (cutoff of >500 reads assigned per bacterial species) and intersection of OTUs detected by all three methods is given in Table S7. Because of the high stringency that allowed only unique paired-end reads with >97% identity to be considered, the specI method for microbial identification yielded only 50 OTUs assigned to the species-level. The dbAssign algorithm, which employs coverage of reads uniquely mapped across prokaryotic genomes, identified 316 bacterial species. Results of rapsearch2 analysis, employing relaxed stringency (DNA-protein comparison using blastx), indicated 1,655 species. The number of microbial species assigned in Figure S10 reflects both size and diversity of the respective databases. The specI method used a compilation of 1,753 prokaryotic species clusters^34^ whereas the dbAssign toolset used a database consisting of 5,614 prokaryotic genomes that had been fully sequenced and curated. The rapsearch2 method used the complete and non-redundant NCBI protein database. The number of OTU assignments is therefore a function of mapping stringency as well as reference database size and curation.

To assess false positive identifications, the results of all three methods were compared, yielding a shortlist of 33 bacterial species identified by all three methods. Table S7 lists the species of these 33 microbes. Strain-level identification was provided by the specI mappings that cluster all reference genomes into species groups based on the similarity of 40 universal marker genes. The cross-validated set of 33 species identified in blowfly and housefly microbiomes were categorized according to the habitat from which they have been isolated and assigned to the potential disease association record of the Pathosystems Resource Integration Center (PATRIC)^35^. Three species are associated with insects, including different species of the endosymbiont *Wolbachia* spp. and *Bacillus thuringiensis* (Bt). The latter produces crystal proteins used as a biopesticide for insect pest control of genetically modified crops. The Bt strains identified in the fly samples are most closely related to serovar chinensis CT-43, toxic for moths, butterflies, and flies^36^. The remaining of the 33 species are either environmental isolates or have been described as associated with plants, animals or humans.

Potential disease association indicated that the pathogens identified in the fly microbiomes may colonize plants, animals and humans. Three species of *Pectobacterium* related to soft-rot disease were identified. These bacteria are necrotrophs, infecting a range of hosts, degrading the cell wall and promoting the death of host cells. They are reported to consume cellular nutrients and degrade plant tissues, turning the host plant to liquid mush^37^. *P*. *atrosepticum* almost exclusively infects potatoes^38^ and was identified as being associated with the Brazilian fly samples by rapsearch2. Brazil is the second largest producer of potatoes in South America, with cultivation restricted mainly to the southern and southeastern regions of the country where most of the fly samples were collected (Food and Agriculture Organization of the United Nations - FAOSTAT, 2008).

*Aeromonas salmonicida*, an animal-associated species, was identified in the housefly microbiome associated with flies collected at a farm in Brazil where there was a lake used for recreational fishing. *A*. *salmonicida* is a salmonid fish pathogen causing furunculosis, a bacterial septicaemia, even at low infectious dose^39^. *Lactococcus garvieae*, also a fish pathogen, is the etiological agent of lactococcosis^40^, a form of hyperacute, haemorrhagic septicaemia in fishes that was initially described as affecting Japanese yellowtail (amberjack), but later detected also in fishes in Mediterranean and Eastern countries. Interestingly, *L*. *garvieae* was identified as present only in significant amounts (>1000 reads) in blowflies collected in Singapore near a canteen (samples SGP_1593, SGP_AJ63, SGP_AJ65, SGP_AJ67 and SGP_AJ68).

The 33 species that had been identified by all the methods employed in this study (Table S7) also comprised opportunistic pathogens of humans. Commonly described as of environmental origin or as a component of the normal skin or gut flora, these bacteria have been reported to cause a variety of nosocomial infections, including diarrhea, septicemia, bacteremia, pneumonia, skin and soft tissue infections, osteomyelitis, and urinary tract infections. For example, *Acinetobacter* spp. notably have been reported as nosocomial pathogens with long-term survival in hospital environments and transmitted directly between patients or via fomites^41^.

Potential human pathogens identified in the blowfly and housefly microbiomes are species associated with both nosocomial and generic infections, such as bacteraemia, septicaemia, and gastroenteritis. In a few cases, these species are etiological agents of specific disease, like in the case of erysipelas (in animals) and erysipeloid (in humans) caused by *Erysipelothrix rhusiopathiae* ^42^. Similarly, *Proteus mirabilis* identified in the microbiomes of the blowfly and housefly is often associated with urinary tract infections^43^. It has also been reported to be present in the salivary glands of the green bottle fly, *Lucilia sericata*, and the larvae of the screwworm fly, *Cochliomyia hominivorax*, interestingly as producing antibiotic molecules^44^. *P*. *mirabilis* generates volatile swarming signals in decaying proteinaceous material that induces attraction and oviposition in flies^45^. The odorant that is produced is hypothesized to serve as a lure for carrion-feeding flies, enabling transport and dispersal of the bacteria.

**Figure 5:**
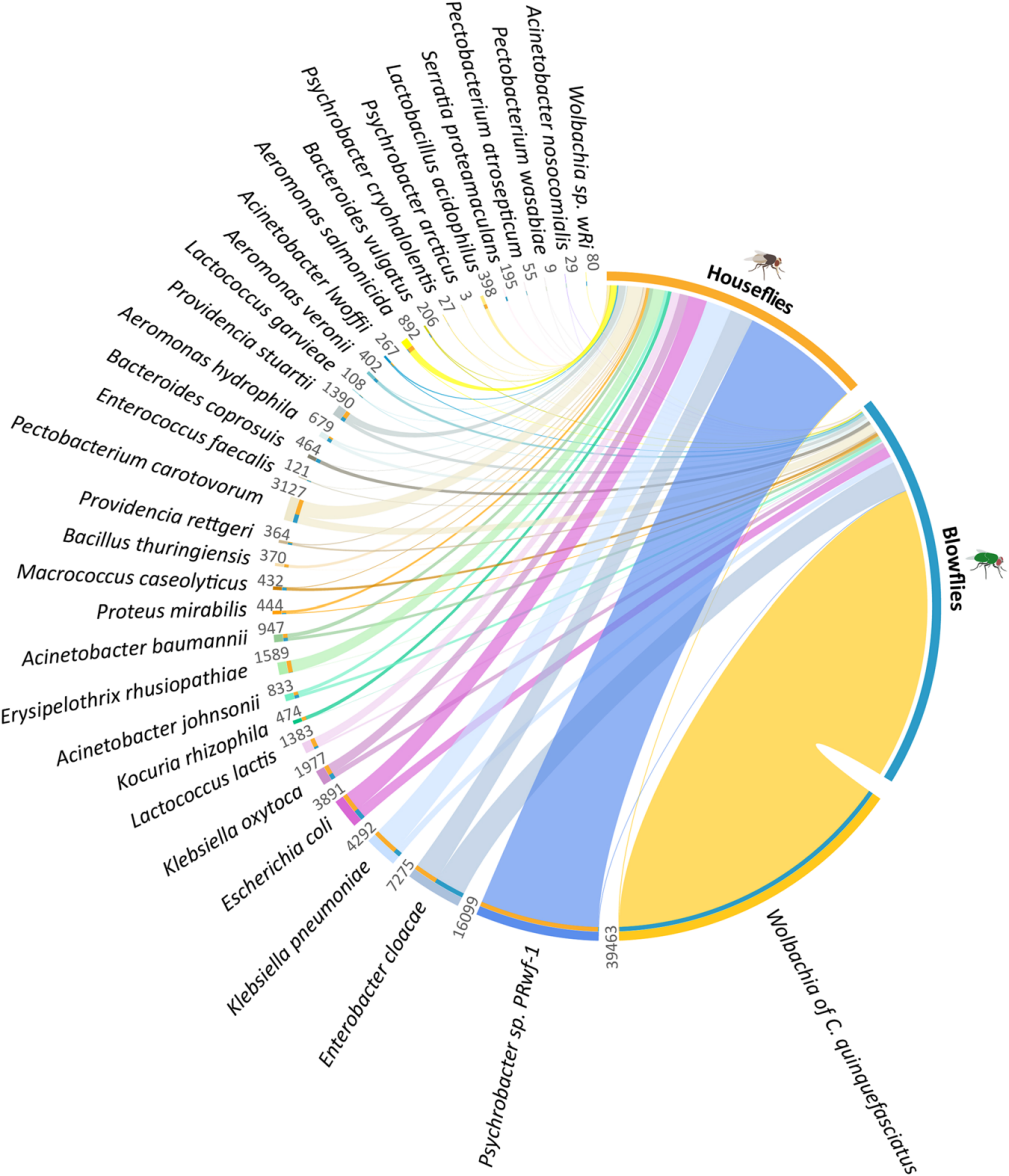
Occurrence and abundance of OTUs in blowflies and houseflies shows both shared and specific microbiomes. Circular visualization of the distribution of 33 bacterial species identified by all three bioinformatics methods. Thickness of the ribbon refers to number of reads of each bacterial species assigned to a fly host by dbAssign. The outer ring summarizes the total number of reads assigned to each bacterial species analysed (values in grey) whereas the relative abundance of reads present in the fly hosts is represented in the innermost ring by orange (housefly) and blue (blowfly) bars. Part of the microbiome is shared by both fly species (environment-acquired), and part is species specific (host-selected). See Table S7 for information about disease-association of 33 bacterial taxa identified by all methods. Data was parsed with Circos table viewer^72^.

Clearly, the opportunistic and the potentially pathogenic bacteria identified in the blowfly and housefly microbiomes are not necessarily associated with a clinical condition or infection of a specific host, whether animal, plant, or human. Rather, it is important to note that mechanical vectors disperse a range of bacterial species to a variety of hosts. The risk of infection ultimately depends on host susceptibility and contact with the agent transported by the insect vector, which moves from one reservoir to another. Figure 5 shows occurrence and abundance of the 33 microbial species identified by all of the bioinformatics methods (Table S7) used in this study and for all fly samples collected, showing host-specific species as well as the shared microbiome of both the housefly and the blowfly. The host-specific patterns of distribution for the *Wolbachia sp*. and for *Psychrobacter sp*. PRwf-1, together with the more extensive incidence of *E*. *cloacae*, *E*. *coli*, and *P*. *carotovorum*, suggest that the microbiomes of carrion-feeding flies represent a combination of host-inherited microorganisms and those acquired from a similar habitat and/or migrations.

### The microbiome of fly body parts

**Figure 6:**
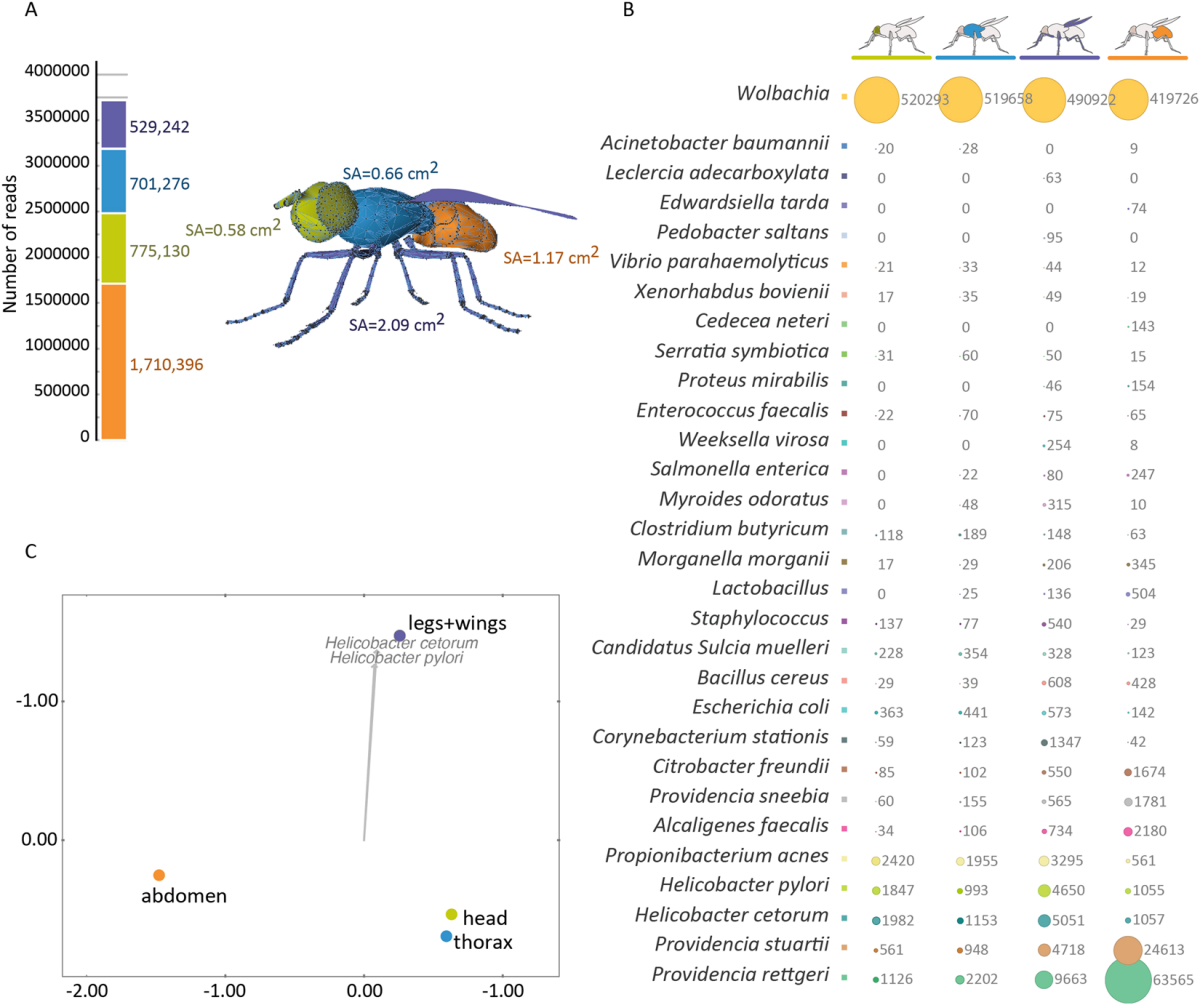
Microbiome of the four body parts. (**A**) A 3-dimensional model of a fly (composed of 11,421 triangles) was used to calculate the approximate surface areas (SA) of four body parts, indicating that the legs + wings, despite small volume and mass, have the largest surface area. The stacked bars indicate total number of reads for each dissected body fraction, showing the abdomen generated more reads than other body parts, most likely because it contains the major part of the intestinal tract and other organs. (**B**) Bubble chart of the top 30 bacterial species assigned to each of the four body parts of a blow fly. The endosymbiont *Wolbachia* sp. was detected in all tissues. Despite the tiny amounts of biological material recovered from legs + wings, the microbial diversity of this body part was found to be higher than other tissues. The bubble chart indicates presence of *Helicobacter* spp. in the blowfly, most prominently in legs + wings. This could be a potential route of dispersion of this pathogen to humans and animals. See Figure S11 for alpha-diversity of the four body parts, Table S8, and Figure S13 for results obatained with Cosmos ID metagenomics software package. (**C**) PCoA of the microbiome of the head, thorax, abdomen and legs + wings, showing separation of legs + wings dataset is mainly driven by presence and abundance of *Helicobacter* species. See Figure S12 for genome-wide coverage of *H*. *pylori* with metagenomics reads of the four body parts. PCoA was generated using Bray-Curtis ecological index (stress = 0.80).

Contamination of surfaces upon which the fly lands and traverses occurs by both physical and passive mechanisms, and thus a mechanical vector can potentially infect susceptible humans, animals, and plants. Most studies to date have investigated the gastrointestinal tract of insects, without addressing the role of the outer body of flies. It can be hypothesized that the fly feet, wings, mouthparts and other body surfaces constitute the main route of microbial dispersal by mechanical vectors. Therefore, we investigated the bacterial abundance and diversity of four distinct body parts of a blowfly: head, thorax, abdomen, and legs + wings. Based on a computer-generated three-dimensional model of a fly, the relative ratio of surface areas (SA) of a blowfly was estimated to be 1.0:1.1:2.0:3.6 for head, thorax, abdomen, and legs + wings, respectively (Fig. 6A). The abdomen accounted for more than twice the number of reads generated relative to other body fractions, likely because this body part covers most of the gastrointestinal tract. Despite a small body mass, the legs + wings fraction yielded the highest diversity of bacterial species (Figure S11) and likely plays a significant role in dispersing bacteria by the fly from one landing site to another.

The bubble chart in Fig. 6B shows the 30 species of bacteria most frequently associated with a given body part. The endosymbiont *Wolbachia* sp. is the most abundant and ubiquitous organism for all body parts of the blowfly. The fact that all body parts have shown similar abundances of *Wolbachia* spp. is an indirect evidence of somatic localization of this endosymbiont in the blowfly *C*. *megacephala*. This suggests that not only germline cells are being colonized, but somatic tissues as well^46^.

The microbiomes of the four body parts are essentially similar to the range of diversity previously described for the 62 blowflies in Fig. 3, indicating that most of the microorganisms associated with flies are not restricted to their gastrointestinal tract. In fact, the PCoA of the four body parts, seen in Fig. 6C, indicates that the microbiome of the head and thorax are more similar to each other, while the microbiomes of the abdomen and legs + wings clustered separately. *Providencia* spp. are more abundant on the abdomen and have been isolated from whole insects and from the gut of blowflies, stable flies, houseflies and fruit flies, also showing a varied virulence and mortality in *D*. *melanogaster* ^47^ by being capable of avoiding their detection by the insect immune system^48^. Interestingly, the dissimilarity that drives the separation of the microbiome of the legs + wings is the abundance of *Helicobacter cetorum* and *Helicobacter pylori* (Fig. 6B and C). Both species are closely related and their proteomes showed an average similarity of >75%^49^. It is estimated that *H*. *pylori* and *H*. *cetorum* diverged 450,000 years ago^50^ and are part of the same branch together with *H*. *acinonychis* ^51^.

### Detection of *Helicobacter pylori*

The surprisingly high incidence of *Helicobacter pylori* motivated us to explore the presence of this important human pathogen on the blowfly body in greater detail. *H*. *pylori* is known to colonize the human stomach and chronic infection with *H*. *pylori* can result in peptic ulcers, increased risk of mucosa-associated lymphoid tissue lymphoma, and even gastric adenocarcinoma^52^. Transmission of *H*. *pylori* and its colonization of humans are not fully understood, but oral-oral and fecal-oral routes of transmission have been proposed. Houseflies were reported to be an alternative reservoir, after it was shown that *H*. *pylori* survives in the gut of houseflies artificially infected in the laboratory^53^. However, the presence of virulent strains of *H*. *pylori* captured from the natural environment has never been reported. In the study reported here, several individual wild-caught blowflies were found to carry *H*. *pylori* DNA. The *H*. *pylori* genome coverage was evaluated using genome-wide assignment methods. Figure S12 shows 5,890 reads of DNA extracted from body parts which mapped across the *H*. *pylori* reference genome (NC_000915.1). A total of 408,693 bp were covered, with an average pairwise identity of 97.5%. The reads are distributed across the genome, covering 25.3% of the reference sequence. Specific virulence factors were identified, showing that both the *cag* pathogenicity island (cytotoxin-associated genes) and *vac*A gene (vacuolating cytotoxin autotransporter) are present. The *cag* pathogenicity island is responsible for unique virulence factors of *H*. *pylori* strains with enhanced virulence^54^, while *vac*A is a multi-functional toxin with polymorphisms linked to increased risk of gastric cancer^55^. Further analysis was performed using the Cosmos ID metagenomics software package as described previously^56^, which identified *H*. *pylori* with relative abundance of 0.57% on the abdomen, 0.6% on the thorax, 2.29% on the head, and 7.21% on the legs + wings (Figure S13 and Table S8). In addition, CosmosID detected four virulence factors associated with *H*. *pylori*, including *cag*11, *cag*18, *flg*C, and *fur* (Figure S13).

*Helicobacter* sp. reads were found in 15 blowflies of the 116 sampled in this analysis and all had been collected in Brazil. Most likely, the flies acquired this bacterium from untreated, open sewage sites or outside latrines. Whether *H*. *pylori* survives and persists on the outer body of the fly has not been determined. However, *H*. *pylori* remains viable for up to 12 hours on agar plates exposed to air and can be ingested and excreted by houseflies infected in laboratory conditions^53^. Also, the acidic pH of 2.9–3.5 of the midgut of blowflies^57^, may play a role in the viability of *H*. *pylori*. These findings strongly indicate further study of alternative routes of transmission of *H*. *pylori* is needed, notably those that may be mediated by flies in both urban and rural environments.

### Mechanisms of dispersal

**Figure 7:**
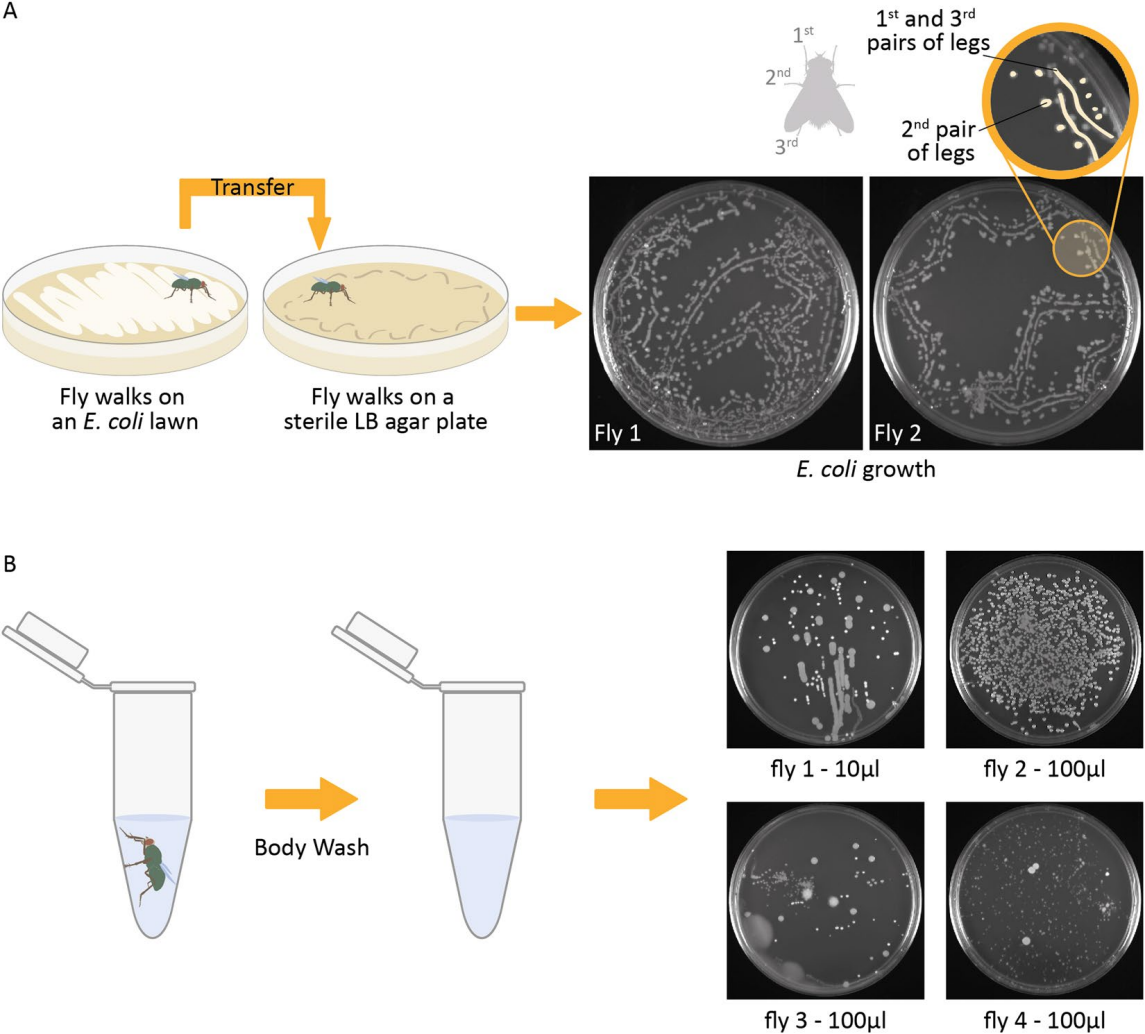
Microbial transport by a mechanical vector. (**A**) Blowflies were exposed to a Petri dish with an *E*. *coli* lawn and then allowed to walk on a fresh, sterile agar plate. The path of the fly walking on the agar can be seen as footprints after incubation. The line of growth indicates that dispersal of bacteria by the blowfly occurs mainly via the legs. The track pattern matched the arrangement of the three pairs of legs, with the first and the third pair resulting in nearly continuous, linear bacterial growth and the second pair of legs generating separate circular colonies on the outside of the lines (see inset for detail). In a few instances, it was possible to observe bacterial growth between the lines of growth probably from mouthparts. (**B**) Growth experiments to evaluate viability of bacteria on the outer body surface of blowflies. The flies were sampled in urban environments near a food court and washed with PBS sterile buffer for 10 minutes. The buffer was then spread in LB agar plates and incubated at 37 °C overnight. Up to 30,000 CFUs were estimated from body washes, showing that bacteria can be transported on the exterior surfaces of flies in a viable state that they can be cultivated.

It has been reported that flies transmit microorganisms by regurgitation, excretion, and via contact with a body part^58^. To investigate mechanisms of bacterial transmission, blowflies were introduced into a Petri dish and allowed to land on an *E*. *coli* lawn (marker strain) that had been inoculated onto LB agar. Subsequently, the flies walked across the surface of sterile agar plates and the pattern of *E*. *coli* observed on the plates, after incubation, matched the footprints of their walk (Fig. 7A). This simple experiment, not unique to our study, shows clearly that flies can disperse bacteria from one landing site to another and that the inoculum can persist over time and after a series of many individual contacts. Unique to this experiment was that there was little evidence of bacterial growth consistent with contact of the abdomen or mouthparts of the fly with the agar, indicating that legs of flies are the main source for rapid and extensive dispersion of bacteria. Blowflies and houseflies show anatomical features, such as bristles and pads, coated with substances that increase the adhesion of particles to their legs and feet^59^. These structures maximize surface area and may promote retention of bacteria that can then be dispersed to other surfaces. By sequencing the DNA extracted from bacterial colonies on agar from the footprints of the fly, we verified that the *E*. *coli* strains at the point of landing and beginning of the fly walk on agar were identical (data not shown). These results demonstrate the vector capacity of flies for bacterial dispersal and show that flies can transport viable, cultivatable bacteria from one place to another, thus confirming the DNA-based evidence discussed above. Further evidence of bacterial viability on the outer body of a fly was verified with bacterial growth from sterile buffer used for washing individual flies collected in urban environments. The washes were spread on LB agar plates and yielded up to 30,000 colony-forming units (CFUs) per fly (Fig. 7B). However, the epidemiological relevance of these findings will depend on determining that viability of bacteria on the body of flies persists over extended periods of time and/or if those bacteria have a significant role in the host life cycle, as has been demonstrated for *P*. *mirabilis* ^45^.

### Intersection of synanthropic microbiome datasets

It is notable that a portion of the blowfly and housefly pan-microbiomes overlaps (Figure S14) with the gastrointestinal microbiome of humans^60^ and the urban microbiome^61^, emphasizing the role of carrion flies as a shuttle between source and sink that are ecologically associated with humans and animals. The precision of our taxonomic identification of bacterial species allows the conclusion that carrion flies are a proxy for the environment from which a significant part of their microbiome is acquired. It highlights the importance of surveillance of fly microbiomes, especially in densely populated areas. If included in public health surveillance programs, it will be possible to predict and prevent routes of transmission of microbes and potential pathogens mediated by these mechanical vectors.

## Conclusions

By sequencing the holobiomes of individual insects, we demonstrate that houseflies and blowflies contain a host-specific microbiome. Nevertheless, a significant shared microbiome, comprising approximately half of the bacterial species, crosses the host species boundary and is present in the microbiomes of the two carrion-flies. As described for biological vectors, our data suggest that flies also harbour a stochastic component in their microbiome, serving as mechanical vectors of bacteria largely derived from decaying material in the environment. Lastly, it is also concluded that adhesion of bacteria from the environment to the outer surface of the exoskeleton, notably the legs and wings, is a significant route of transmission of bacteria from surface to surface, including plant, animal, and human pathogens. The environmental sequencing approach presented in this study can be an effective tool for vector control programs and public health environmental surveillance.

## Materials and Methods

### Sampling

Blowflies (*C*. *megacephala*) and houseflies (*M*. *domestica*) were collected with an entomological net using decomposing fish as bait. Flies were immediately placed in individual microcentrifuge tubes and maintained on dry ice for further taxonomic identification and photodocumentation. All samples were stored at −80 °C until DNA extraction was performed.

Flies were collected from urban, rural and natural sites in three countries: Brazil, the United States, and Singapore. Urban sites included a food market, the emergency entrance of a public hospital, a public park in a metropolitan setting, a sanitary landfill for household waste, and a hawker centre/food court. The natural environment comprised of a protection area at the Instituto Nacional de Pesquisas Amazônicas in the Amazon rainforest. Rural sites included a mixed farm with animal stables for pigs, horses, chickens, and cattle, and a poultry farm. Table S2 lists the location, coordinates, and metadata associated with the 116 samples analysed in this study.

### Blowfly colony

A laboratory strain of the blowfly *C*. *megacephala*, derived from a colony maintained for 20 generations at the Laboratory of Animal Evolutionary Genetics (University of Campinas, Brazil), was reared as an environmental control. One female fly was kept in a cage with mouse carcass to stimulate oviposition. After the eggs hatched, larvae were reared to pupae in mouse carcasses provided by the animal facility at University of Campinas and maintained under controlled conditions (temperature 25 °C ± 1 °C; 70% relative humidity; 12/12 hour photoperiod) in a rearing chamber Fitotron (Eletrolab, model EL011). Pupae were individualized in tubes and kept in the same conditions until the emergence of adults. Ninety-eight adult flies newly emerged were pooled and their DNA was used as an environment control.

### DNA extraction and sequencing

Total DNA of individual flies was extracted with DNeasy Blood & Tissue kit (Qiagen). For the blowflies, the individuals were usually split (if weighing >25 mg) and two separate DNA extractions were performed for each individual. After DNA precipitation, the DNA from two extractions was combined in a single tube. For houseflies, usually weighing <25 mg, the DNA extraction was performed with an entire, individual fly. One individual blowfly was also dissected into head, thorax, abdomen and legs + wings using bleached fine-tipped forceps and a sterile scalpel. The total DNA was then extracted from each of the four body parts. Some modifications were made to the DNA extraction protocol suggested by the manufacturer. Briefly, flies (or body parts) were ground and homogenized with a microcentrifuge teflon pestle in ATL (animal tissue lysis) buffer containing 20 µl of Proteinase K. The mixture was vortexed and incubated at 56 °C overnight to complete the tissue lysis. For maximum DNA yield, elution from the column was conducted twice with 50 to 100 µl of AE buffer (provided with the kit). DNA samples were quantitated by Qubit fluorescence assay (Thermo Fischer) and DNA integrity was determined on a 2100 Bioanalyzer (Agilent Technologies). Total DNA was sheared to 300 bp using a Covaris S220 focused-ultrasonicator (Covaris Inc.). Automated library construction was either performed on a SPRI-TE Nucleic Acid Extractor (Beckman Coulter), using the SPRIworks Fragment Library Kit I or on a Bravo NGS Workstation (Agilent Technologies) with Illumina TruSeq DNA sample preparation reagents, following the manufacturers’ recommendations. For all library preps barcoded, TruSeq DNA LT adapters (Illumina Inc.) were used to enable library pooling for sequencing. For samples processed on the SPRI-TE, the DNA was size-selected on a Pippin Prep (Sage Science) electrophoresis system prior to library preparation to remove small DNA fragments. The size-selection was performed on 2% agarose ethidium free cassettes (Sage Science), followed by manual DNA purification with Agencourt AMPure XP magnetic beads (Beckman Coulter). Finished libraries were quantitated with Quant-iT^TM^ Picogreen^**®**^ (Invitrogen) and concentrations validated by qPCR, following KAPA SYBR® FAST qPCR kit instructions (Kapa Biosystems). Equimolar amounts (8–12pM) of libraries were pooled for multiplexed sequencing on a HiSeq. 2000 or HiSeq. 2500 (Illumina Inc.) platform with 101-bp, 151-bp or 251-bp paired-end protocols. Table S2 shows the read length produced for each sample. Sequencing was performed at the Singapore Centre for Environmental Life Sciences Engineering, Nanyang Technological University (Singapore) and at the Center for Comparative Genomics and Bioinformatics, The Pennsylvania State University (University Park, PA, USA).

### Metagenomic analyses

Raw fastq files were trimmed for both adapter and low-quality bases using cutadapt 1.0^62^. A maximum error rate of 0.2 during the search for the adapter sequences was allowed, and a quality cutoff of 20 was used to trim low-quality ends from reads before adapter removal. High-quality reads were aligned against an in-house draft genome of *C*. *megacephala* (unpublished) and the *M*. *domestica* reference genome^63^ (AQPM00000000.1) to filter out the host reads, thus reducing the size of datasets and the computational resources required for the metagenomic analyses. The alignments were done using bowtie2 v2.1.0^16^ with parameters selected for higher level of sensitivity. The remaining non-host fraction of the datasets was used for the individual microbiome analyses of 62 blowflies, 53 houseflies, and 1 pool of lab-reared flies used as environmental control. The non-host sequence reads used for metagenomic analyses are available under NCBI BioProject ID PRJNA385554.

The resulting non-host filtered metagenomic datasets were analysed with three separate methods: (i) alignment of the translated reads against the non-redundant (NR) database using rapsearch2^64^; (ii) alignment of reads against a customized prokaryotic genomic database using dbAssign; and (iii) alignment of short-read datasets against specI representative genomes^34^. Specific bit-score cutoffs were implemented for read assignment to microbial taxa, based on the read length (Table S2).

The rapsearch2 (v2.15) analyses were performed with the nucleotide fastq format as input in a multithreaded run. Reads were translated in six frames and aligned against the NCBI NR protein database (downloaded on 12 April 2015) with BLASTX using default parameters.

dbAssign is an in-house python toolset (https://github.com/aakrosh/dbAssign) that can be used to assign fragments to multiple reference sequences from a customized database. We created a database that included 5614 prokaryote genomes available on NCBI (downloaded on 25 February 2016 - complete and chromosome-level genomes). Reads were aligned against the database using BWA v0.7.4^65^ with the default parameters to find all alignments that exceeded a score threshold. dbAssign was used to filter and keep matches with a minimum coverage of 90% of the bases and a minimum identity of 95% over those bases. dbAssign then permutes all likely alignments of the fragment using the filtered alignments of the reads and assigns the fragment to a particular reference if both reads of the fragment can be aligned to it. It calculates a bit-score for the alignments and the details are output in the BLAST tabular format. NCBI taxonomy IDs were recorded for use as an input to MEGAN 5^66^, which was restricted to show only unique paired-read matches assigned to a species-level bacterial taxon. Matches to multiple ‘species’ were assigned to the higher taxonomical level.

For the alignment of reads against a database of 1,753 representative genomes, we used specI (http://vm-lux.embl.de/~mende/specI/)^34^. This method groups reference genomes into species clusters, based on 40 universal, single-copy marker genes and allows for species-level identification. The microbial fraction of paired-end reads were competitively mapped using BWA v0.7^65^, using default parameters. Reads were counted only when they were mapped with greater than 97% identity. We computed horizontal and vertical coverage per sample over each of the genomes using qaCompute (https://github.com/CosteaPaul/qaTools).

The alignment results were imported into MEGAN 5 and taxa assignments were done with strict parameters of the LCA-assignment algorithm and considering the read length generated for each sample (Table S2), with the following settings: Max Expected = 0.01, Top Percentage = 10.0, Min Support = 25, Min Complexity = 0.33, Paired Reads = On.

All metagenomic analyses were performed for individual datasets and further normalized to perform comparative analyses of microbiomes. The datasets were normalized to the dataset with the smallest number of reads of the 116 samples to represent relative abundances of assigned bacterial taxa and presented as a bubblechart in Fig. 3. The bubbles were plot with a log-squared scale. The calculation of principal coordinate analysis (PCoA) was performed using Bray-Curtis dissimilarity^67^ and the tree was generated with UPGMA method to show hierarchical clustering of fly species based on their microbiome. Species diversity index Shannon-Weaver^68^ was calculated using ‘species’ level of NCBI taxonomy to generate a table with MEGAN 5^66^. PERMANOVA and ANOVA statistical analysis were performed with phyloseq.^69^, as implemented in the tool MicrobiomeAnalyst^70^. Venn diagrams were generated with BioVenn^71^ using the ‘species’ level table as an input to generate area-proportional representation.

The alignment of assigned reads against full reference genomes of the *M*. *domestica* Salivary Gland Hypertrophy Virus (NC_01067) and *Helicobacter pylori* (NC_000915.1) was conducted using bowtie 2^16^ with default parameters.

### Bacterial growth

Individual blowflies were collected and enclosed in a chamber with a Petri dish containing an LB-agar plate covered with a lawn of *Escherichia coli* K12. The flies walked on the lawn for about 30 seconds before the plates were exchanged with a fresh sterile LB agar plate. After the flies had walked on the sterile plate for 30–60 seconds, they were incubated overnight at 37 °C and photographed. To investigate the presence of bacteria on the outer body of flies, sixteen whole adult blowflies were incubated in sterile PBS buffer at room temperature for 10 minutes with gentle shaking. Volumes of 1, 10 and 100 µl of the fly body washes were plated on LB-agar Petri dishes and incubated at 37 °C overnight. Plates with colony-forming units were counted and photographed.

### Data and software availability

The full datasets used for metagenomic analyses (non-host datasets) were deposited in the SRA database and are available under NCBI BioProject ID PRJNA385554.

The source code for dbAssign is available at https://github.com/aakrosh/dbAssign. dbAssign is a new python toolset developed to assign short DNA fragments to multiple reference sequences from a customized database.

## Electronic supplementary material

Supplementary Materials

Supplementary Table S2

Supplementary Table S6

Supplementary Tables S3, S4 and S5
