# Supplementary material for: The microbiomes of blowflies and houseflies as bacterial transmission reservoirs
Source: Sci Rep. 2017 Nov 24;7:16324. doi: 10.1038/s41598-017-16353-x (PMC5701178; doi:10.1038/s41598-017-16353-x)
Supplement: Supplementary file 1 — Supplementary Materials [file 41598_2017_16353_MOESM1_ESM.pdf]

Supplementary Materials

The microbiomes of blowflies and houseflies as bacterial transmission reservoirs

Ana Carolina M. Junqueira, Aakrosh Ratan, Enzo Acerbi, Daniela I. Drautz-Moses, Balakrishnan N. V. Premkrishnan, Paul I. Costea, Bodo Linz, Rikky W. Purbojati, Daniel F. Paulo, Nicolas E. Gaultier, Poorani Subramanian, Nur A. Hasan, Rita R. Colwell, Peer Bork, Ana Maria L. Azeredo-Espin, Donald A. Bryant, Stephan C. Schuster

Figures S1 to S14

Tables S1, S7, and S8

Supplementary data - Tables S2, S3, S4, S5, and S6

Legends for the supplementary excel format tables:

Supplementary Table S2. Metadata of 116 samples of blowflies and houseflies and details of the data generated, filtered, and assigned by each bioinformatics method. Bit-score cutoffs for taxonomic assignment based on read length are also provided.

Supplementary Table S3. Species-level assignment of the microbiome of blowflies (*C. megacephala*) and houseflies (*M. domestica*) using dbAssign. Detailed information about number of normalized reads assigned to each bacterial species in each fly sample.

Supplementary Table S4. Species-level assignment of the microbiome of blowflies (*C. megacephala*) and houseflies (*M. domestica*) using rapsearch2. Detailed information on number of normalized reads assigned to each microbial species in each fly sample.

Supplementary Table S5. Species identification of the microbiomes of blowflies (*C. megacephala*) and houseflies (*M. domestica*) using specI. Detailed information about number of normalized reads assigned to each microbial species in each fly sample.

Supplementary Table S6. Summary of the bacterial species assigned by the dbAssign toolset, indicating shared and unique species in blowflies and houseflies.

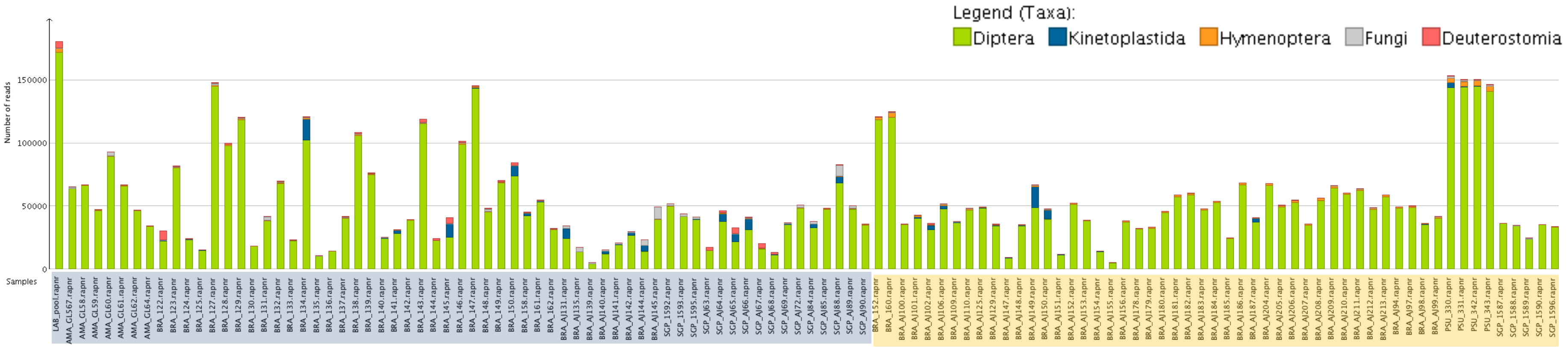

Supplementary Figure S1. Categories of reads assigned to Eukaryotes domain using rapsearch2 against the non-redundant NCBI database, showing that most matched to fly species of the order Diptera, thus revealing the reads that passed host-genome filtering. Blowflies are highlighted in blue and houseflies in yellow.

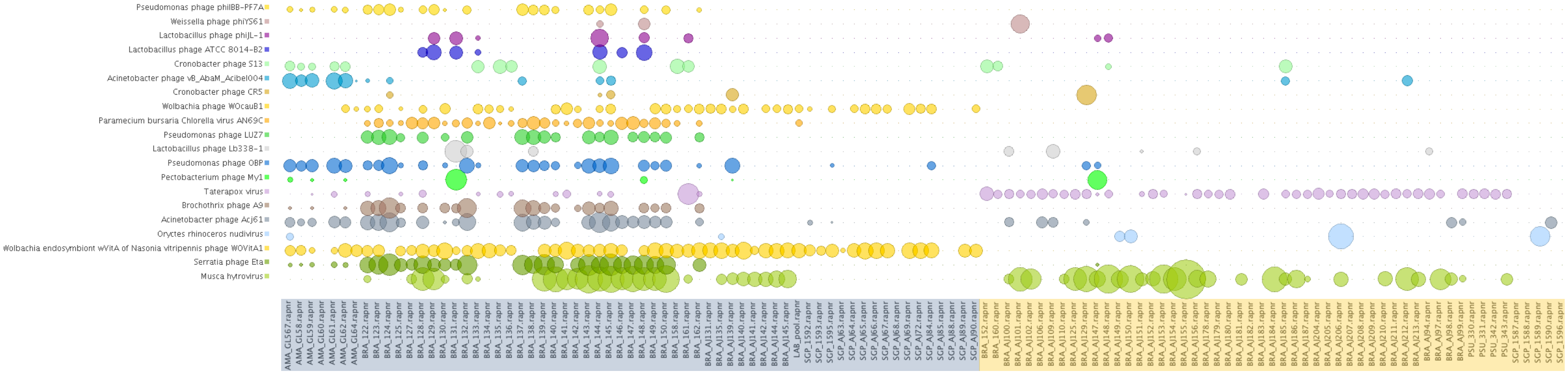

Supplementary Figure S2. The most frequently identified twenty viruses assigned from metagenomics datasets of 116 blowflies and houseflies using the rapsearch2 against the non-redundant NCBI database. Blowflies are highlighted in blue and houseflies in yellow. The bubble size is log-scaled and refers to the normalized number of reads.

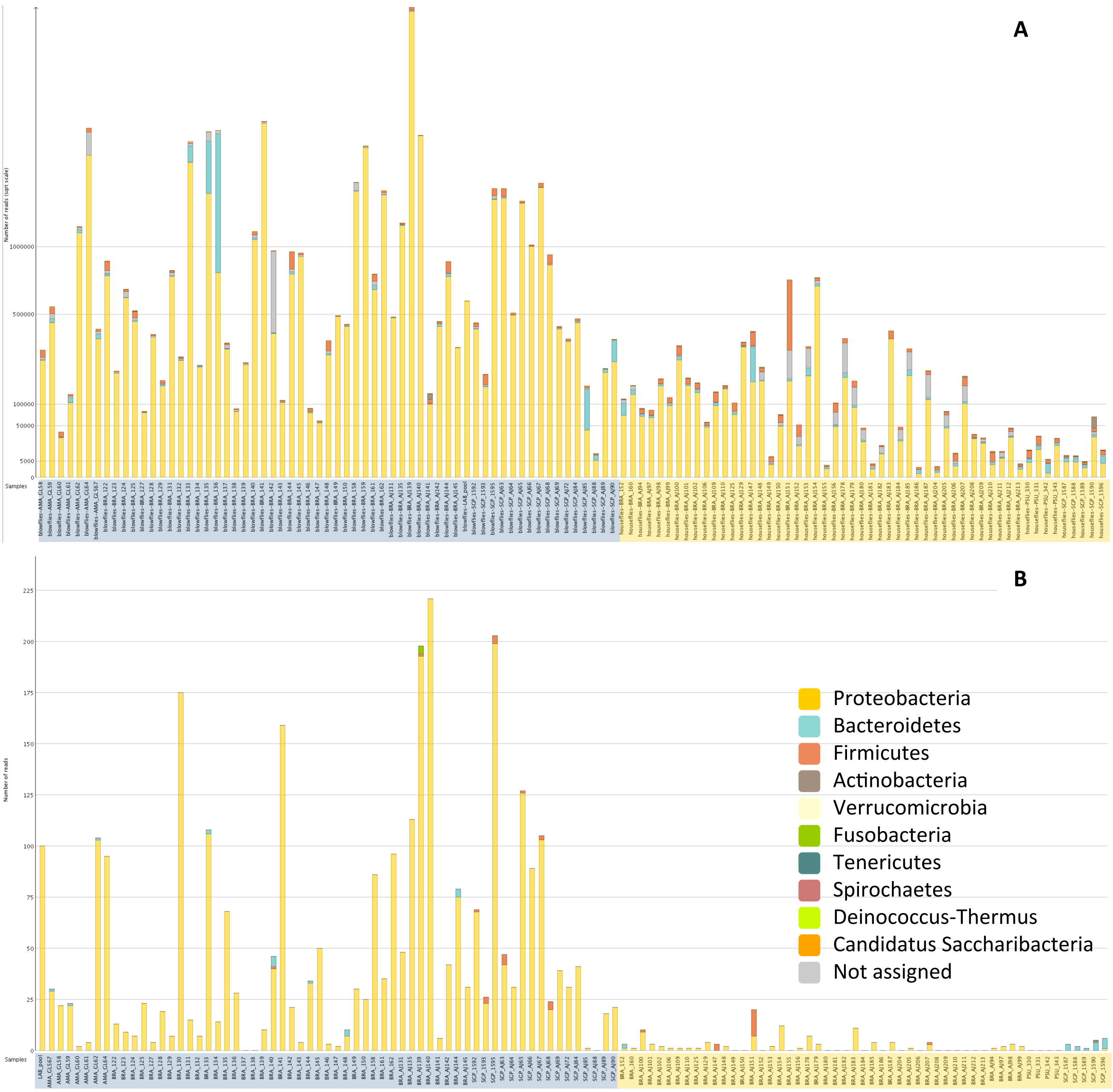

Supplementary Figure S3. Phylum-level assignment of normalized metagenomic datasets with different bioinformatics methods. (**A**) reads assigned against the complete bacterial genomes database from NCBI using in-house developed script dbAssign. (**B**) reads assigned using specI clusters of 40 single universal genes. Proteobacteria, Bacteroidetes and Firmicutes are the most abundant phyla described in the blowfly *Chrysomya megacephala* (blue shadow) and in the housefly *Musca domestica* (yellow shadow).

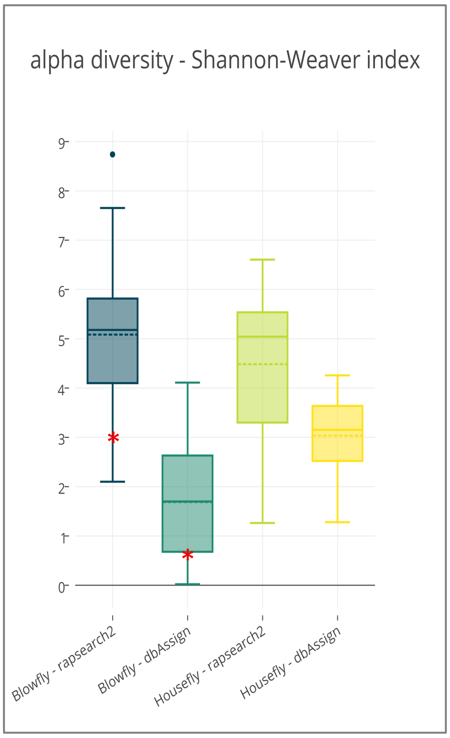

Supplementary Figure S4. Diversity of bacterial taxa in blowflies and houseflies. Boxplots show distribution of alpha-diversity (Shannon-Weaver index) found in 116 samples. Solid line refers to median and dashed line to mean values. Whiskers represent minimum and maximum values. Individual points represent outlier and asterisk indicates alpha-diversity of the lab-reared pool of blowflies serving as control (values of 3.004 for rapsearch2 and 0.576 for dbAssign). Full data are available in Supplementary Tables S2 and S3. Different databases used for rapsearch2 (nr) and dbAssign (bacterial complete genomes) provided different bacterial diversity for blowflies and houseflies. dbAssign recovered less diversity (blowflies=1.69; houseflies= 3.15) than rapsearch2 (blowflies=5.18; houseflies= 5.04) due to different stringencies used for read mapping parameters and different databases.

A

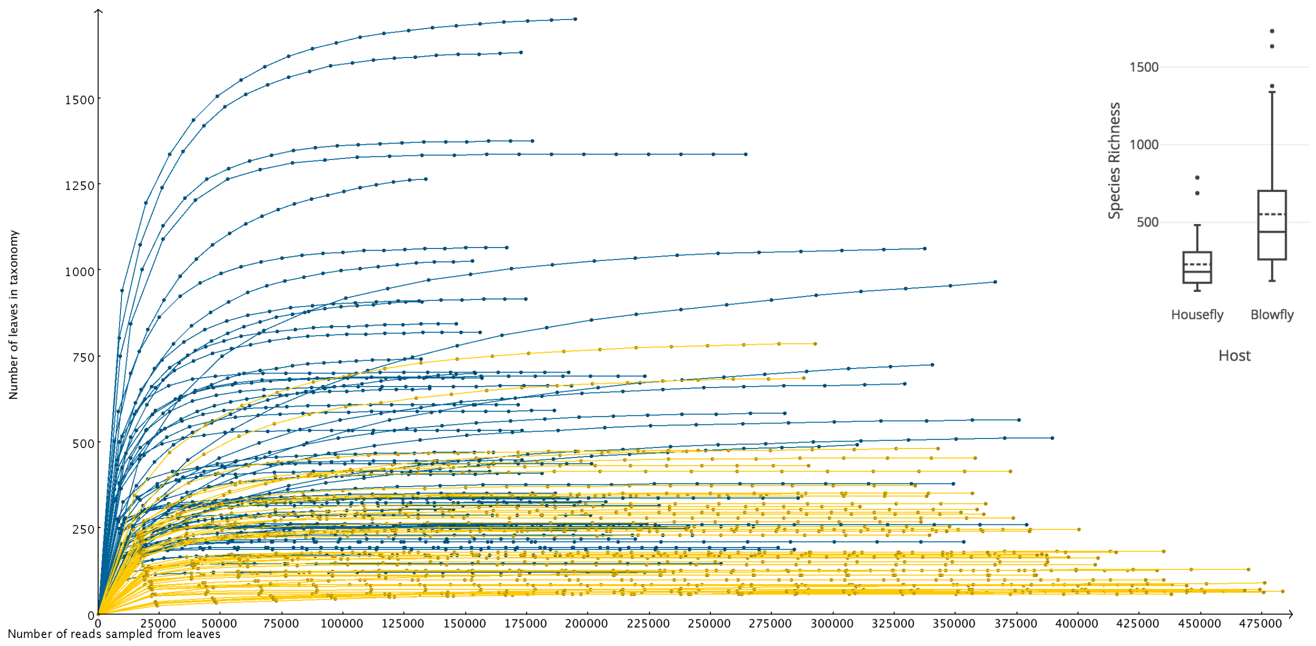

B

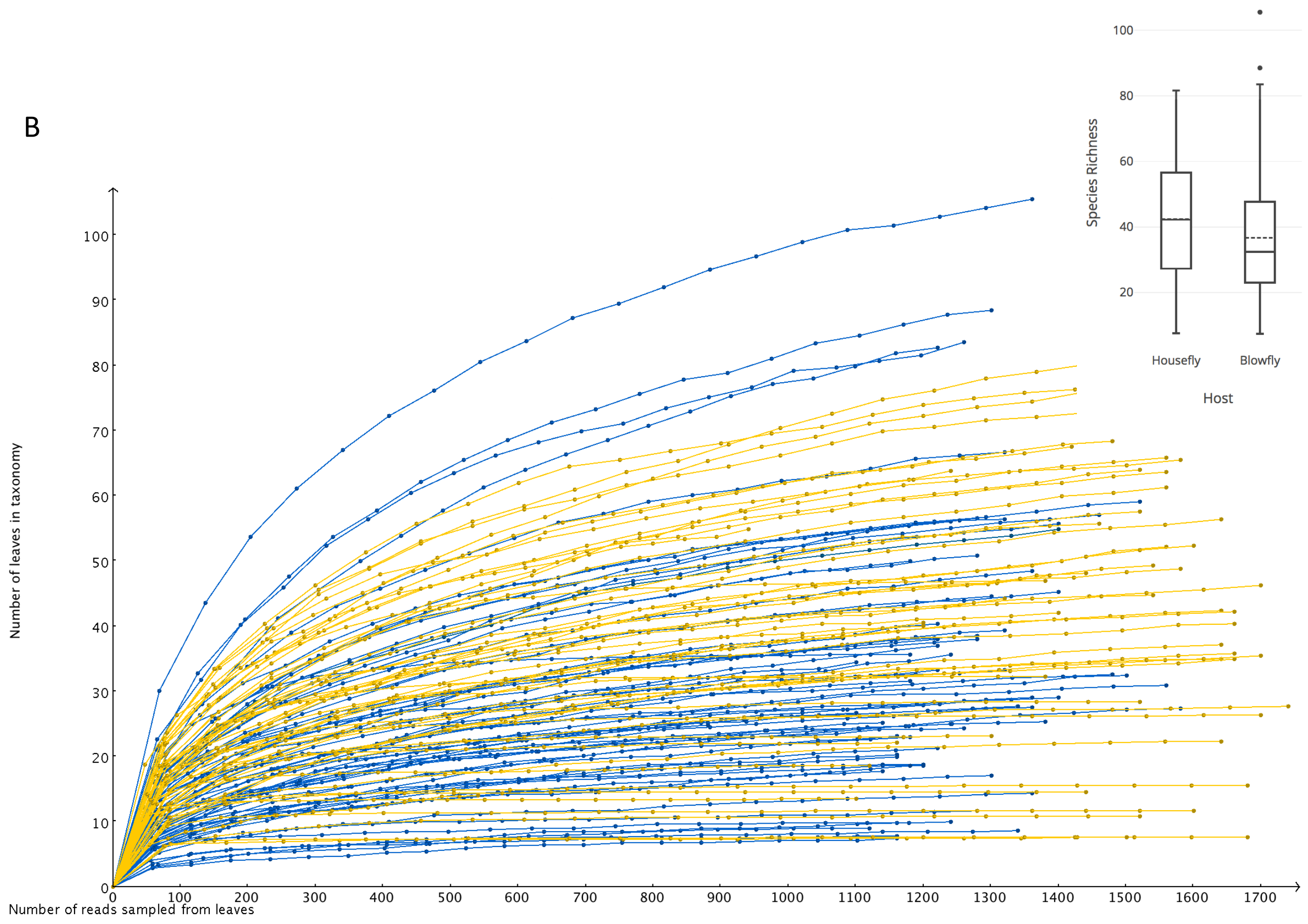

Supplementary Figure S5. Rarefaction curves of all blowflies (blue lines) and houseflies (yellow lines) analyzed with rapsearch2 **(A)** and dbAssign **(B)**. The OTU discovery levels off around 25,000 reads sampled with rapsearch2 and around 500 reads for dbAssign, reflecting differences in databases used and alignment stringency. The boxplots in the right upper corners shows the OTU richness per host species, compiling the total number of observed bacterial species assigned for the two vectors. Solid line refers to median and dashed line to mean values. Whiskers represent minimum and maximum values. Individual points represent outliers.

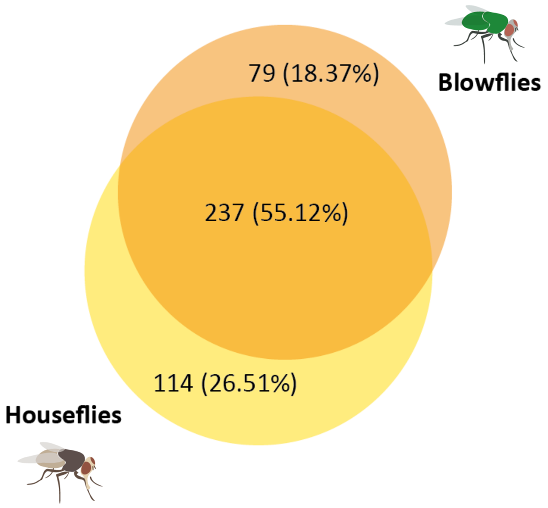

Supplementary Figure S6. Venn diagram indicating that more than 50% of the microbiome of houseflies and blowflies is shared.

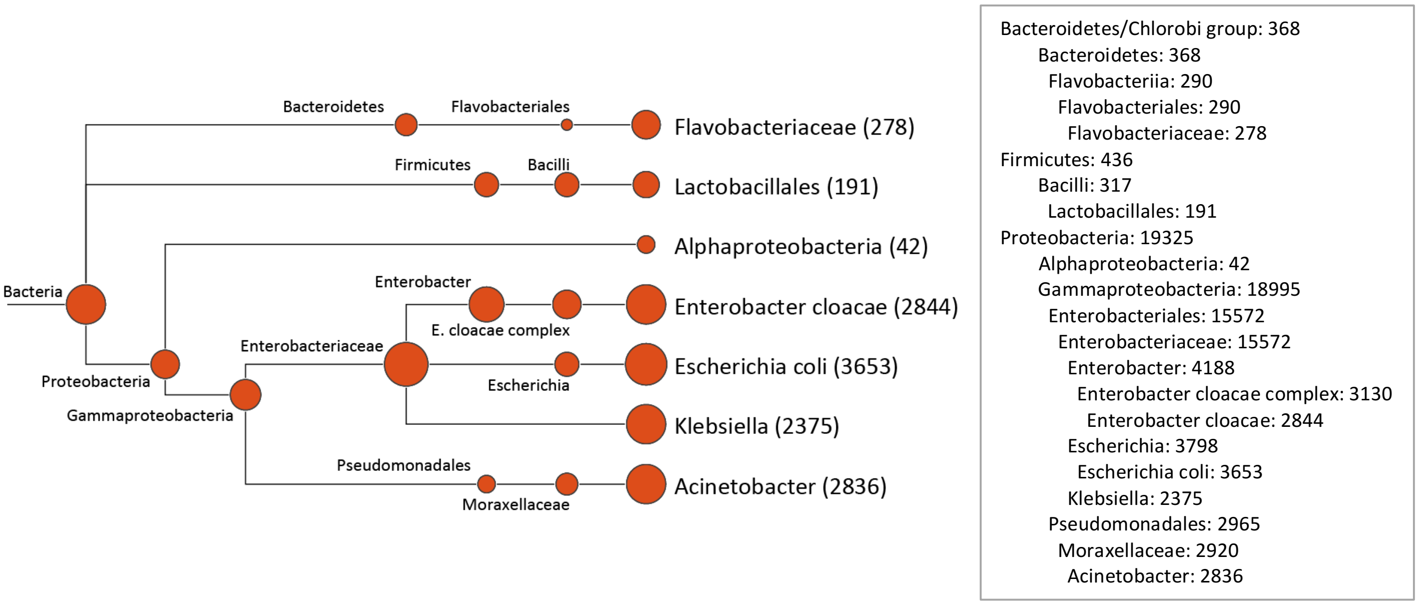

Supplementary Figure S7. The core microbiome of *C. megacephala* and *M. domestica* (dbAssign taxa assignment output). All taxa listed refer to those present in more than 80% of flies analysed. The number of reads assigned to terminal nodes are inside the brackets. Bubble size indicates abundance of reads at different taxonomic levels (log-scaled) and the box describes all reads assigned to different taxonomic levels in the core microbiome.

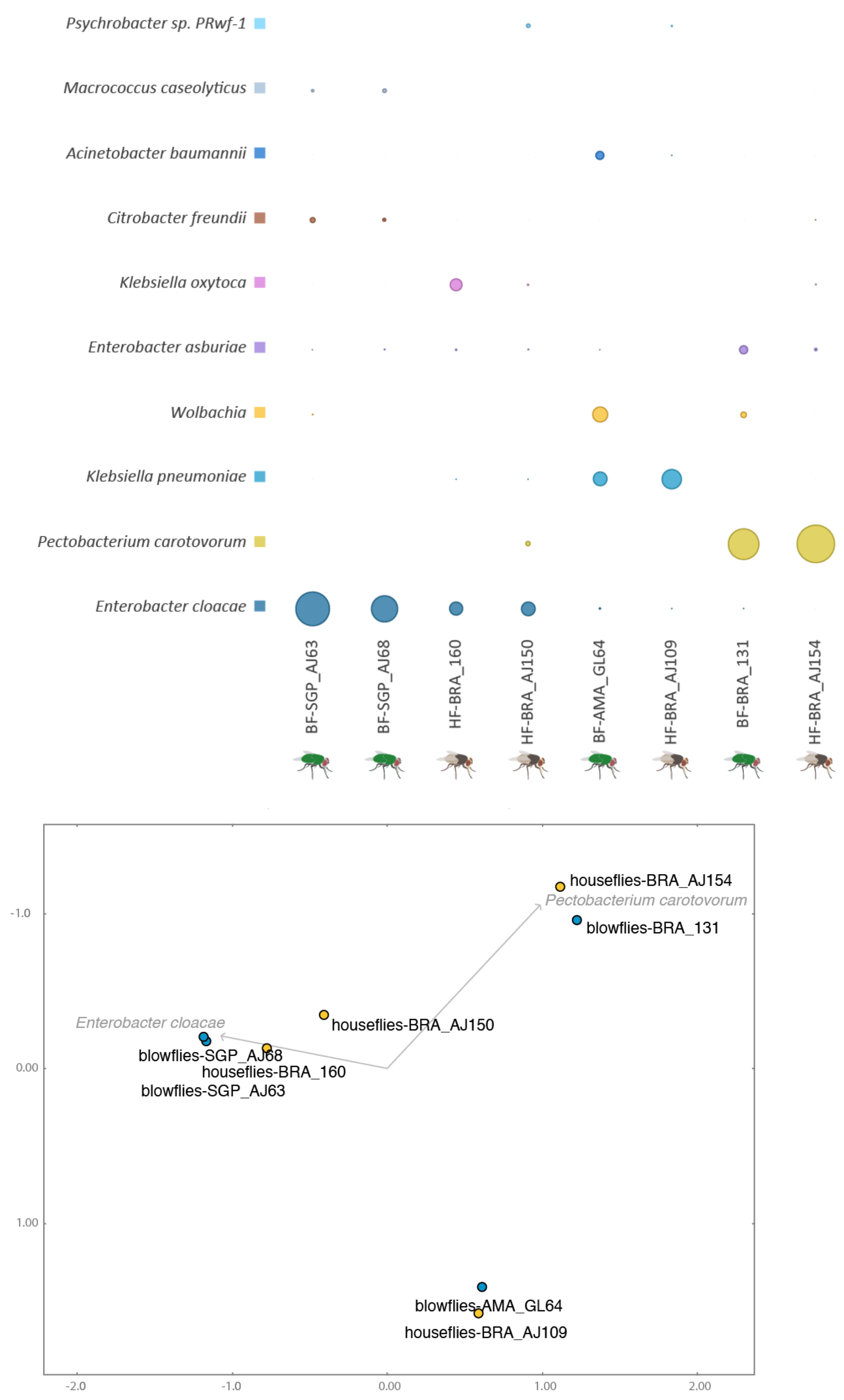

Supplementary Figure S8. Top 10 bacterial species of blowfly and housefly samples that could not be separated with clustering analyses based on their full microbiomes. The blowflies show a low amount of *Wolbachia* spp. compared to other blowfly samples and higher amounts of *E. cloacae*, *K. pneumoniae* or *P. carotovorum*. The PCoA shows main factors driving clustering of these samples.

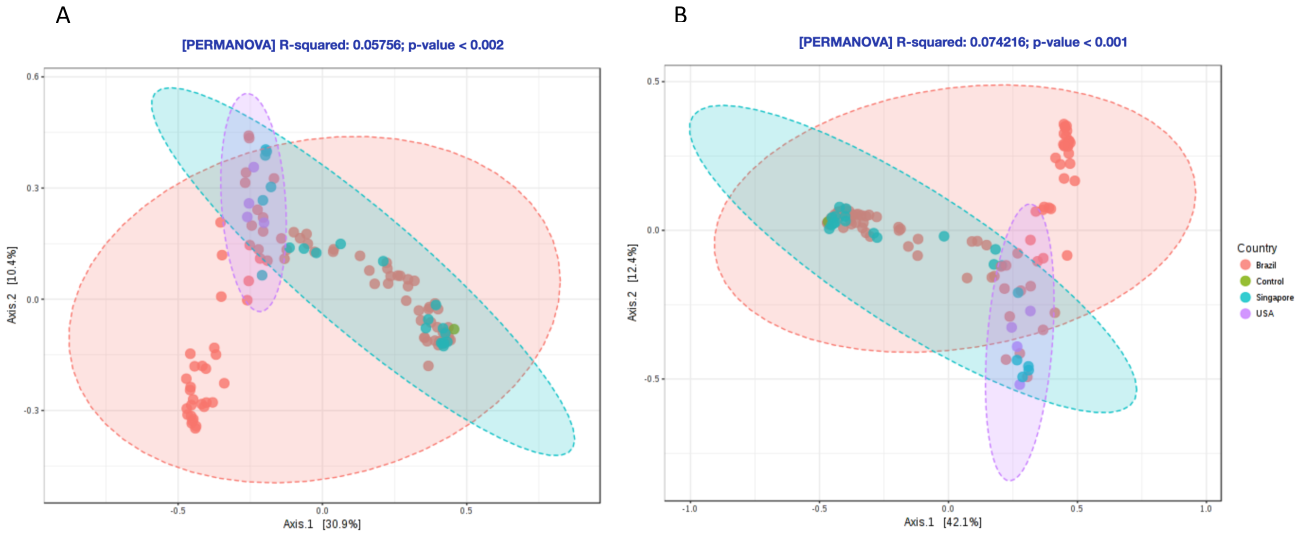

Supplementary Figure S9. PCoA plots of the beta-diversity of microbiome of individual blowflies and houseflies. Samples are coloured based on geographical origin of the samples. The geographic origin of samples had a minor effect on the segregation of flies and explains a small proportion of the beta-diversity variation found with rapsearch2 (**A**) and dbAssign (**B**). R-squared and p-values are shown above the plots. PCoA and PERMANOVA were generated with the normalized datasets using 'species' taxonomic rank with Bray-Curtis index.

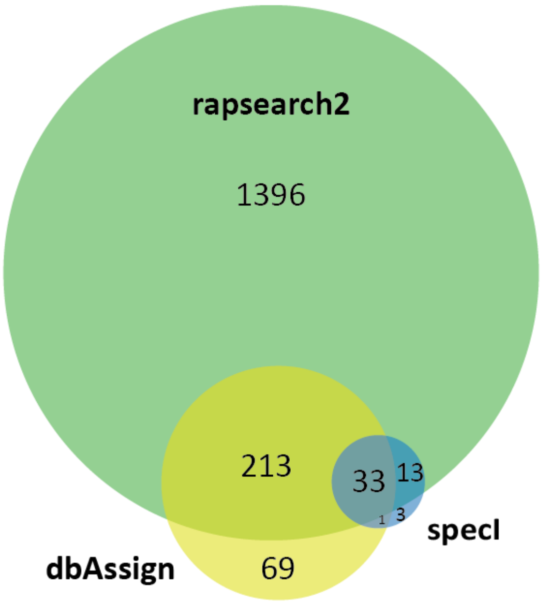

Supplementary Figure 10. Comparison of methods used for OTU assignment. Venn diagram showing the number of OTUs assigned to species by three different bioinformatics approaches. Only high-confidence paired-end reads that were mapped above 97% identity are represented by the specI circle (total of 50 OTUs) and only OTUs with more than 500 mapped reads were considered for analysis with rapsearch2 (1,655 microbial species) and dbAssign (316 prokaryotic species) comparison. A total of 33 bacterial species were identified by all 3 methods and are listed in Table S7.

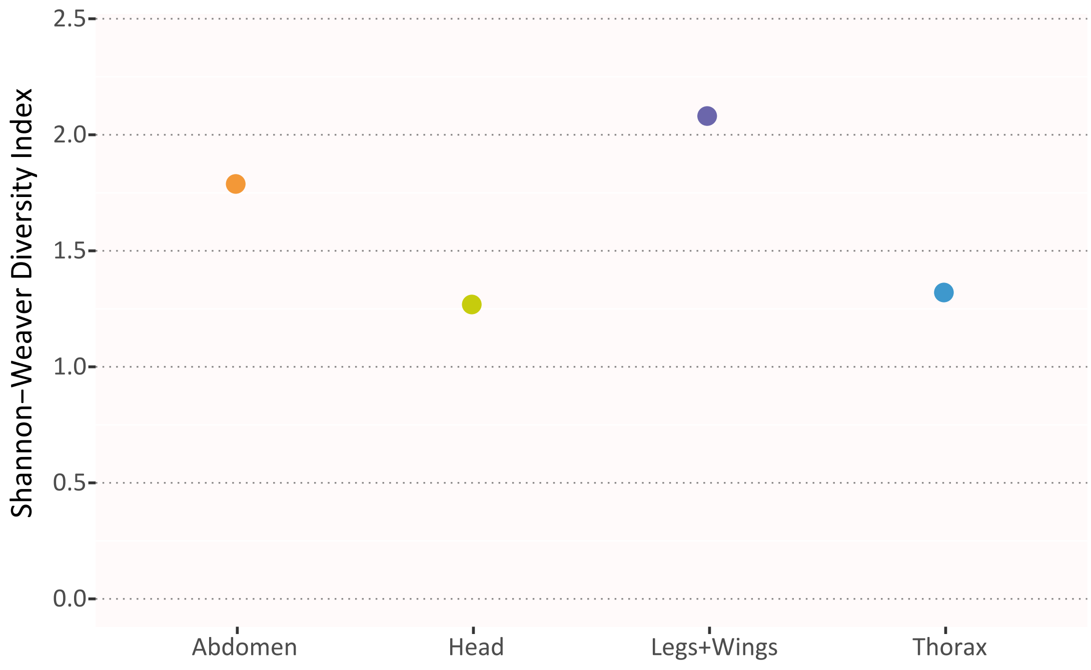

Supplementary Figure S11. Alpha-diversity of bacterial species assigned to the microbiome of four body parts of the blowfly (Shannon-Weaver diversity index). Legs+wings harbor the highest bacterial diversity, despite the least number of reads generated, compared to head, thorax and abdomen. This is particularly important in establishing the role of the outer body as the main route of bacterial dispersal by mechanical vectors. Fungi, archaea and viruses show a low diversity and were not detected in most of the flies analyzed, as well as in the four body parts.

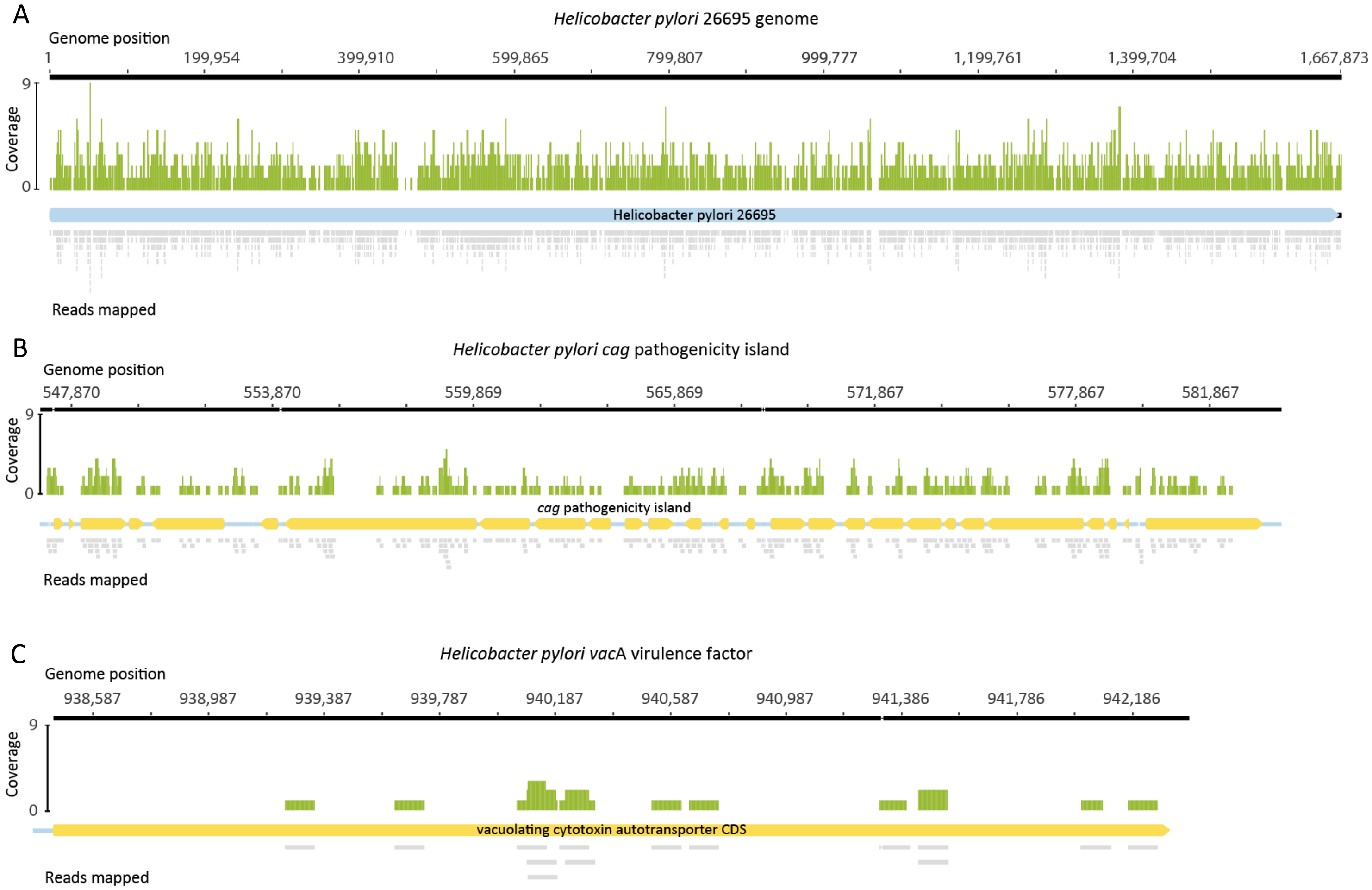

Supplementary Figure S12. *Helicobacter pylori* genome coverage by metagenomic reads. **(A)** Reads assigned to the genus *Helicobacter* spp. were extracted from the four body parts in the dbAssign output and mapped against *H. pylori* strain 26695 reference genome (NC_000915.1). A total of 5,890 reads were mapped using bowtie 1.1.2 (default parameters), covering 25.3% of the reference genome. **(B)** Metagenomic reads covering the gene cluster in the *cag* pathogenicity island. A total of 18,281 identical sites were mapped with pairwise identity of 97.9%. **(C)** Metagenomic reads covering the *vac*A virulence factor of *H. pylori*. The length of the region analysed is 3,019 bp (partial *vac*A gene), with 966 identical sites covered with pairwise identity of 95.6%.

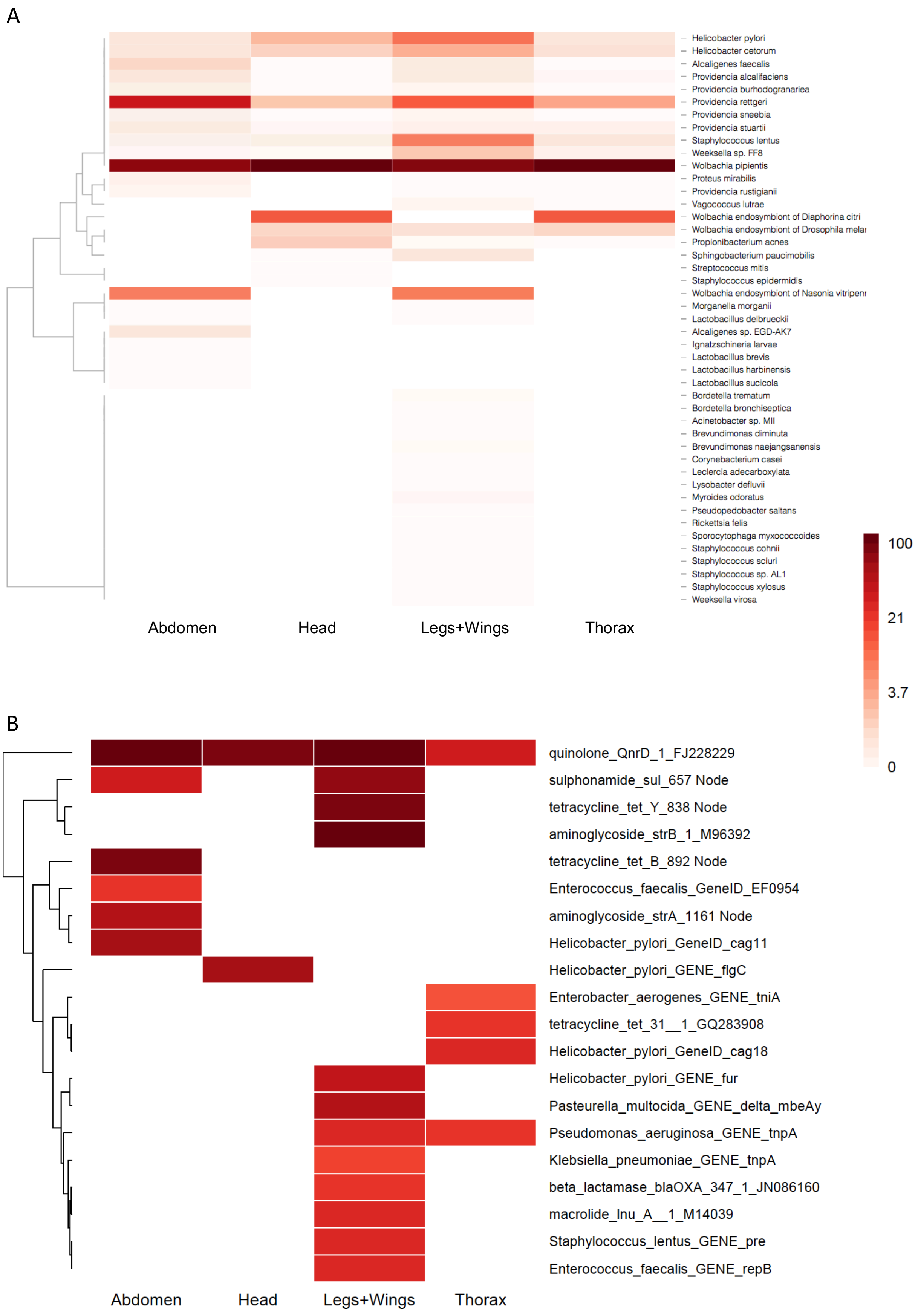

Supplementary Figure S13. (A) Metagenomic and **(B)** virulence factors screening of the four body parts using CosmosID metagenomics software package. Both analyses corroborate the dbAssign findings and also indicate presence of *H. pylori* in the four body parts of the fly, mostly concentrated in the legs+wings tissues.

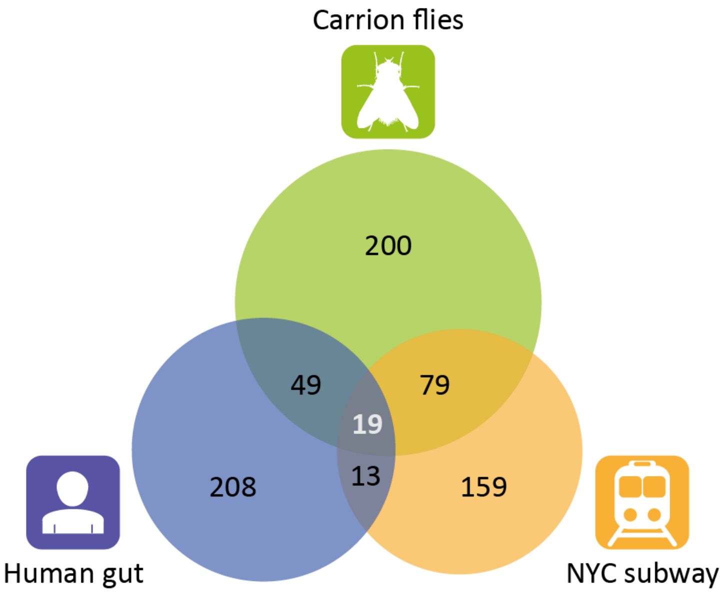

Enterobacter cloacae
Klebsiella pneumoniae
Escherichia coli
Proteus mirabilis
Leuconostoc mesenteroides
Providencia rettgeri
Lactobacillus plantarum
Clostridium perfringens
Enterococcus faecalis
Enterococcus faecium
Lactobacillus brevis
Lactobacillus delbrueckii
Streptococcus infantarius
Eubacterium rectale
Lactobacillus helveticus
Lactobacillus paracasei
Alistipes shahii
Lactobacillus casei
Micrococcus luteus

Figure S14. Overlapping of bacterial species among the carrion flies microbiomes, human gut microbiomes project (data from the gastrointestinal tract downloaded from http://hmpdacc.org/catalog/) and the urban microbiome conducted in the New York City subway system (NYC subway). The highlighted table lists 19 bacterial species that overlap all three microbiome projects.

Supplementary Table S1. Summary of the average number of reads generated and assigned per sample. The lab pool containing 98 individual flies reared in a controlled environment was not included in the summary**.**

**Fly species**	**Average number of reads per sample**				
	Total of reads	Non-host reads	Assigned by rapsearch2	Assigned by dbAssign	Assigned by specI
Blowfly	69,980,118.4	25,990,875.2	1,503,825.2	812,787.9	50.0
Housefly	44,834,740.9	25,888,798.7	343,073.0	115,935.7	2.3

Supplementary Table S7. List of 33 bacterial species identified by all three bioinformatics methods used for comparative analyses of the microbiome of 116 individuals of blowflies and houseflies. The use of specI as representative of species clusters allowed for identification of bacterial species and strains, enabling further investigation of potential sources of transmission and disease association (PATRIC database).

Species identification (3 methods)	Strain identification (specI)	Habitat	PATRIC disease association	Host association
Bacillus thuringiensis	*Bacillus thuringiensis* serovar chinensis CT-43	Insects (mainly dipterans and lepidoterans)	None	
Wolbachia Culex quinquefasciatus	*Wolbachia* endosymbiont of *Culex quinquefas*	Endosymbiont - mosquito	None	Insect-associated
Wolbachia sp wRi	*Wolbachia pipientis* wRi	Endosymbiont - fly	None	
Acinetobacter johnsonii	*Acinetobacter johnsonii* SH046	Soil, water	None; rare nosocomial diseases	
Aeromonas veronii	*Aeromonas veronii* B565	Aquaculture pond sediment	None	
Bacteroides coprosuis	*Bacteroides coprosuis* DSM 18011	Swine feces	None	
Klebsiella oxytoca	*Klebsiella oxytoca* KCTC 1686	Ubiquitous and opportunistic in nature.	None	
Klebsiella pneumoniae	*Klebsiella pneumoniae* 342	Maize	None	
Kocuria rhizophila	*Kocuria rhizophila* DC2201	Rhizosphere of narrowleaf cattail	None	Environmental, plant, animal or human-associated
Lactobacillus acidophilus	*Lactobacillus acidophilus* NCFM	Gastrointestinal tract	None	
Lactococcus lactis	*Lactococcus lactis* subsp. *lactis* KF147	Mung bean sprouts	None	
Macrococcus caseolyticus	*Macrococcus caseolyticus* JCSC5402	Animal meat, cow's milk, bovine organs and food-processing factories	None	
Psychrobacter sp PRwf 1	*Psychrobacter sp.* PRwf-1	Food spoilage	None	
Psychrobacter cryohalolentis	*Psychrobacter cryohalolentis* K5	Permafrost soils	None	
Psychrobacter arcticus	*Psychrobacter arcticus* 273-4	Permafrost soils	None	
Serratia proteamaculans	*Serratia proteamaculans* 568	Plant - Poplar endophyte	Pneumonia (one case reported)	
Aeromonas salmonicida	*Aeromonas salmonicida* subsp. *salmonicida*	Aquatic	Furunculosis in fish	otential fish pathogen
Lactococcus garvieae	*Lactococcus garvieae* Lg2	Fish	Lactococcosis in fish	
Pectobacterium carotovorum	*Pectobacterium carotovorum* subsp. *carot*	Plants (wide host range)	Soft rot	
Pectobacterium atrosepticum	*Pectobacterium atrosepticum* SCRI1043	Potato stem	Soft rot	tential plant pathogen
Pectobacterium wasabiae	*Pectobacterium wasabiae* WPP163	Potato	Soft rot	
Acinetobacter lwoffii	*Acinetobacter lwoffii* SH145	Skin	Nosocomial diseases	
Acinetobacter nosocomialis	*Acinetobacter nosocomilais* RUH2624	Skin	None; nosocomial diseases	Opportunistic pathogens of animals and humans
Bacteroides vulgatus	*Bacteroides vulgatus* ATCC 8482	Human gastrointestinal tract	Peritonitis	
Enterobacter cloacae	*Enterobacter cloacae* subsp. *cloacae* ATC	Human gastrointestinal tract. Also found in water, sewage, soil and food	Nosocomial diseases	
Providencia rettgeri	*Providencia rettgeri* DSM 1131	Water, soil, human gastrointestinal tract	Gastroenteritis	
Providencia stuartii	*Providencia stuartii* ATCC 25827	Oil, water, and sewage	Urinary tract infection, septicaemia	
Acinetobacter baumannii	*Acinetobacter baumannii* TCDC-AB0715	Multiple	Nosocomial diseases, bacteraemia	
Aeromonas hydrophila	*Aeromonas hydrophila* subsp. *hydrophila*	Water	Gastroenteritis, septicaemia	
Enterococcus faecalis	*Enterococcus faecalis* V583	Multiple	Bacteraemia, endocarditis, urinary tract infection	
Erysipelothrix rhusiopathiae	*Erysipelothrix rhusiopathiae* str. Fujisawa	Soil, food scraps and water contaminated by infected animals	Erysipelas, erysipeloid	ntial human pathogens
Escherichia coli	*Escherichia coli* O26:H11 str. 11368	Multiple	Hemorrhagic colitis	
Proteus mirabilis	*Proteus mirabilis* HI4320	Human urinary tract	Encephalitis, urinary tract infection, pyelonephritis,	

Supplementary Table S8. Identification and relative abundance of the bacterial strains in the metagenomes of the four body parts (head, thorax, abdomen and legs+wings) using the CosmosID metagenomics software package. Only those bacterial strains identified with confidence and above threshold are listed.

Body part	Bacterial strain	Unique Matches Frequency	Unique Matches	% Unique Matches	% Total Matches	Relative Abundance
Head	Wolbachia_pipientis_wBol1_b	148046	2179	21.18	55.95	81.22
	Wolbachia_endosymbiont_of_Diaphorina_citri	47849	736	3.28	27.11	10.58
	Helicobacter_pylori_16354 Branch	354	322	10.25	10.31	2.29
	Providencia_rettgeri_DSM_1131	20461	17927	9.11	9.57	1.68
	Propionibacterium_acnes_TypeIA2_P_acn17	14	14	1.81	8.28	1.34
	Helicobacter_cetorum_MIT_00_7128	7370	6069	7.57	7.61	1.2
	Wolbachia_24169 Branch	1476	21	0.64	0.37	1.12
	Staphylococcus_lentus_F1142	2834	2487	1.71	1.70	0.24
Thorax	Wolbachia_pipientis_wBol1_b	127523	2174	21.13	55.89	82.6
	Wolbachia_endosymbiont_of_Diaphorina_citri	41906	706	3.15	26.99	10.58
	Providencia_rettgeri_DSM_1131	34004	27145	13.80	14.45	3.12
	Wolbachia_24169 Branch	1354	25	0.76	0.46	1.11
	Helicobacter_cetorum_MIT_00_7128	3964	3220	4.02	4.11	0.7
	Helicobacter_pylori_16354 Branch	148	133	4.23	4.93	0.6
	Staphylococcus_lentus_F1142	4758	4008	2.75	2.77	0.48
	Wolbachia_24159 Branch	105	5	0.70	0.46	0.23
	Bacteroidetes_Phylum_25 127_597 Branch	6	6	4.14	4.14	0.16
Abdomen	Wolbachia_pipientis_wBol1_b	271025	2219	21.57	56.13	57
	Providencia_rettgeri_DSM_1131	2312538	150809	76.68	78.86	27.14
	Wolbachia_endosymbiont_wVitB_of_Nasonia_vitripennis	24906	348	3.51	44.74	6.11
	Wolbachia_24168 Branch	162	5	0.13	0.13	4.9
	Alcaligenes_faecalis	19221	14677	10.78	17.75	1.08
	Helicobacter_pylori_16354 Branch	397	365	11.62	12.88	0.57
	Providencia_alcalifaciens_205_92	9614	1007	3.89	7.14	0.55
	Helicobacter_cetorum_MIT_00_7128	8879	6966	8.69	8.74	0.54
	Alcaligenes_sp_EGD_AK7	63	47	3.05	15.28	0.52
	Providencia_stuartii_strain_ATCC_33672	20459	675	2.85	1.49	0.4
	Providencia_burhodogranariea_DSM_19968	32261	857	0.44	0.49	0.21
	Staphylococcus_lentus_F1142	5103	4443	3.05	3.05	0.17
	Providencia_sneebia_DSM_19967	23675	816	0.45	0.51	0.17
	Proteus_mirabilis_strain_FDA_MicroDB_86	7389	213	2.01	1.23	0.12
	Providencia_rustigianii_DSM_4541	15081	515	0.26	0.36	0.09
	Proteus_35886 Branch	342	8	0.10	0.53	0.07
	Weeksella_sp_FF8	1766	1598	1.08	1.10	0.06
	Bacteroidetes_Phylum_25 127_597 Branch	6	6	4.14	4.14	0.05
	Morganella_36472 Branch	195	37	0.11	0.24	0.05
Legs+Wings	Wolbachia_pipientis_wBol1_b	90380	2171	21.10	55.89	62.35
	Providencia_rettgeri_DSM_1131	111188	73674	37.46	39.17	10.21
	Helicobacter_pylori_16354 Branch	566	490	15.60	17.10	7.21
	Wolbachia_endosymbiont_wVitB_of_Nasonia_vitripennis	8629	307	3.10	44.50	6.26
	Staphylococcus_lentus_F1142	49647	37882	25.97	26.04	6.09
	Helicobacter_cetorum_MIT_00_7128	12894	10226	12.75	12.83	2.61
	Weeksella_sp_FF8	13355	11498	7.77	7.95	1.61
	Wolbachia_24169 Branch	795	23	0.70	0.40	0.66
	Sphingobacterium_paucimobilis_HER1398	5810	5417	2.76	2.76	0.5
	Providencia_alcalifaciens_R90_1475	703	413	1.50	2.40	0.42
	Alcaligenes_faecalis	1697	1579	1.16	1.92	0.41
	Dietzia_19977 Branch	26	19	2.95	2.95	0.21
